# Melatonin induces drought tolerance by modulating lipoxygenase expression, redox homeostasis and photosynthetic efficiency in *Arachis hypogaea* L

**DOI:** 10.3389/fpls.2022.1069143

**Published:** 2022-12-05

**Authors:** Sharma Shreya, Laha Supriya, Gudipalli Padmaja

**Affiliations:** Department of Plant Sciences, School of Life Sciences, University of Hyderabad, Hyderabad, TG, India

**Keywords:** antioxidants, drought stress, groundnut, lipoxygenase, melatonin, photosynthesis

## Abstract

Melatonin (N-acetyl-5-hydroxy tryptamine), a multipotent biomolecule is well known for its ability to confer tolerance to several abiotic and biotic stresses. The regulation of melatonin-mediated drought tolerance in drought-distinguished varieties can be different due to discriminating redox levels. The present study was focused on assessing the effects of melatonin priming against polyethylene glycol (PEG)-induced stress with respect to the antioxidant system, photosynthetic parameters, lipoxygenase expression, JA and ABA levels in drought-sensitive (Kadiri-7) and drought-tolerant (Kadiri-9) varieties. Exogenous melatonin alleviated the drought stress effects in sensitive variety (Kadiri-7) by increasing the endogenous melatonin content with an improved antioxidant system and photosynthetic attributes. The primed stressed plants of the sensitive variety exhibited reduced expression and activity of the chlorophyll degrading enzymes (Chl-deg PRX, pheophytinase and chlorophyllase) with a concomitant increase in chlorophyll content in comparison to unprimed controls. Interestingly, melatonin priming stimulated higher expression and activity of lipoxygenase (LOX) as well as enhanced the expression of genes involved in the synthesis of jasmonic acid (JA) including its content in drought stressed plants of the sensitive variety. The expression of *NCED3* (involved in ABA-biosynthesis) was upregulated while *CYP707A2* (ABA-degradation) was downregulated which corresponded with higher ABA levels. Contrastingly, priming caused a decrease in endogenous melatonin content under drought stress in tolerant variety (Kadiri-9) which might be due to feedback inhibition of its synthesis to maintain intracellular redox balance and regulate better plant metabolism. Furthermore, the higher endogenous melatonin content along with improved antioxidant system, photosynthetic efficiency and LOX expression associated with the increased levels of JA and ABA in unprimed stressed plants of the tolerant variety (Kadiri-9) is pointing towards the effectiveness of melatonin in mediating drought stress tolerance. Overall, exogenous melatonin alleviated the adverse effects of drought stress in sensitive variety while having no add-on effect on drought stress responses of tolerant variety which is inherently equipped to withstand the given duration of drought stress treatment.

## 1 Introduction

The non-availability of water to plants is a major concern for mankind and also for the whole ecosystem. Global temperature rise and anthropogenic activities including industrialization, deforestation and urbanization affect rainfall patterns (Warner and Afifi, 2014) leading to water scarcity. Drought stress, one of the most limiting factors in agriculture, affects the yield and quality of the crops (Stagnari et al., 2016). Groundnut, an economically important legume crop has attained global importance due to its edible seeds and oil-producing capability (Stalker, 1997). India, the second largest producer of groundnut in the world is estimated to experience a fall in production in 2022 (https://www.statista.com/statistics/769471/india-groundnut-production-volume). Among the various factors that affect its production, abiotic stress especially aridity is a major factor responsible for the decline in its yield worldwide (Carvalho et al., 2017). Therefore, improving the ability of groundnut to withstand drought stress is a primary concern for researchers.

Drought stress adversely affects the growth and development of groundnut plants by interfering with various morphological, physiological and biochemical processes. Lower leaf water potential, turgor pressure with decreased stomatal conductance, photosynthetic and transpiration rate under drought affects the pod development (Reddy et al., 2003). In addition, exposure to drought stress results in the production of reactive oxygen intermediates (ROIs) from photorespiration, photosynthetic apparatus and mitochondrial respiration. The over-accumulation of reactive oxygen species (ROS) in the cells causes oxidative damage by disruption of cellular integrity, inactivation of enzymes and protein oxidation (Mittler, 2002). Plants have developed several defense systems to cope with this oxidative condition *viz.*, non-enzymatic and enzymatic antioxidant pathways (Jung, 2004; Ajithkumar and Panneerselvam, 2014), which scavenge the ROS molecules to maintain cellular redox homeostasis. It is well known that environmental stresses also lead to an increase in malondialdehyde (MDA) which is a lipid peroxidation product, and compatible solutes such as proline and glutamate, which are used as stress markers in plants (Loggini et al., 1999; Fu and Huang, 2001; Ajithkumar and Panneerselvam, 2014).

Various approaches *viz.*, conventional breeding, molecular breeding and genetic manipulation using recombinant DNA technologies have been used to develop drought tolerant varieties in different crop plants. In addition to the above, seed priming with natural and synthetic compounds has proved to be beneficial in improving drought stress tolerance (Jisha et al., 2013). Recent studies suggest that priming of seeds conferred better stress tolerance against adverse environmental conditions by retaining stress memory thereby enabling protection against oxidative stress (Jisha et al., 2013; Ibrahim, 2016; Farooq et al., 2019). Groundnut seeds primed with gibberellic acid exhibited higher antioxidant activity, proline, lesser malondialdehyde and higher chlorophyll content under salt stress (Erbil, 2021). Zinc and iron priming significantly enhanced the yield of field-grown groundnut (Khan et al., 2017). Seed priming with several agents (gibberellic acid, hydrogen peroxide, salicylic acid, ascorbic acid and so on) improved the salinity tolerance of the groundnut plants (Pal et al., 2017).

Melatonin, an amphipathic molecule was first discovered by Lerner et al. (1958) as a skin-lightening agent in animals. It has broadened its horizons to the plant arena due to its ubiquitous nature. In 1993, melatonin was discovered to be a powerful antioxidant and free radical scavenger (Tan, 1993). Melatonin is known to scavenge ROS and reactive nitrogen species (RNS) which are detrimental to the plant system. Exogenous melatonin was found to improve several abiotic stress responses *viz*., salinity, drought, high/low temperature and so on (Debnath et al., 2019). Various studies have also shown a significant role of melatonin in promoting physiological processes under abiotic stresses. Melatonin exhibits a wide variety of effects including enhancement of germination to delayed senescence of plants. Zhang et al. (2020) reported that melatonin improved the germination parameters of seeds and promoted the radicle growth by increasing the antioxidant enzyme activity, balancing cellular osmotic potential and reducing lipid membrane peroxidation against PEG-induced water stress in soybean. Furthermore, transcriptome analysis of melatonin treated *Arabidopsis* revealed altered expression of several defense-related genes indicating its key role in plant defense against various environmental stresses (Weeda et al., 2014). Transgenic plants overexpressing melatonin biosynthesis-related genes have been reported to exhibit delayed leaf senescence (Huangfu et al., 2022).

Phyto-oxylipins, are the products of unsaturated fatty acid metabolism, and its derivatives (*viz.*, JA) are well known to actively participate in plant defense mechanisms (Blée, 2002). Lipoxygenases (LOXs) are a group of enzymes that catalyze the oxylipin formation from polyunsaturated fatty acids (linoleic and linolenic acid) in plants (Blée, 2002; Porta and Rocha-Sosa, 2002). They play important roles in plant development and tolerance to abiotic and biotic stresses (Chen et al., 2022; Mou et al., 2022). LOXs potentially participate in signaling caused by stressor effects and are considered as a stress biomarkers against biotic and abiotic stresses (Singh et al., 2022). Lim et al. (2015) reported that *CaLOX*-overexpressing *Arabidopsis* plants exhibited tolerance phenotypes to drought and high salt stresses *via* rapid scavenging of ROS and by inducing high expression of ABA (abscisic acid) and stress-responsive marker genes. Similarly, the overexpression of *MdLOX* enhanced the salt tolerance in apple calli, and its heterologous expression increased ROS scavenging capacity in Arabidopsis (Chen et al., 2022). Hou et al. (2015) found that *DkLOX3* overexpression in Arabidopsis caused leaf senescence with more lipid peroxidation and ROS accumulation under normal conditions, whereas exposure of such overexpressing lines to high salinity and drought caused a decrease in the accumulation of ROS. It was thus suggested that the participation of LOX in defense pathways is versatile and complicated.

The relationship between melatonin, LOX, JA and ABA is still elusive due to contrasting reports. Luo et al. (2022) reported that alleviation of the alkaline stress by exogenous melatonin in rice seedlings was associated with a decrease in LOX activity and malondialdehyde content. Similarly, the study of Hu et al. (2020) revealed that the protective effects of exogenous melatonin on lateral root formation in response to copper stress in melon seedlings were due to decreased ROS damage as a consequence of reduced expression of LOX-related genes and JA levels. Alharbi et al. (2021) observed that melatonin application alleviated the salt-stress-induced decline in growth by decreasing ROS levels, lipid peroxidation, electrolyte leakage and lipoxygenase activity in seedlings of *Glycine max* (L.). Intriguingly, Radogna et al. (2009) showed the pro-radical effects of melatonin mediated by LOX as they were prevented by a set of LOX inhibitors. Recently, Guo et al. (2022) showed that melatonin induced the accumulation of hydrogen peroxide (H_2_O_2_), accompanied by upregulation of melon respiratory burst oxidase homolog D (*CmRBOHD*) and (Ca^2+^)_cyt_ signaling to offset ABA action to delay leaf senescence in melon (*Cucumis melo* L.).

LOX performs a crucial role in the synthesis of jasmonates, which are involved in diverse physiological processes including plants’ stress tolerance (Ahmad et al., 2016). LOX-induced JA in turn activates MYC2, a positive regulatory transcription factor for the expression of the anti-oxidant defense genes (Ruan et al., 2019). Xing et al. (2020) showed that *CmLOX10* positively regulates drought tolerance through JA-mediated stomatal closure in oriental melon (*Cucumis melo* var. makuwa Makino). There are also reports showing that exogenous application of JA imparts drought tolerance in sensitive cultivar of wheat (Abeed et al., 2021) and different Brassica species (Alam et al., 2014). Jasmonic acid (JA) was found to enhance the ABA accumulation under drought stress (de Ollas et al., 2015). In general, the level of ABA usually increases during abiotic stress conditions and elevated ABA can enhance plant adaptation to various abiotic stresses (Tuteja, 2007). Li et al. (2016) reported that the early drought priming induced an increase in endogenous melatonin production, in the drought-primed plants than in the nonprimed plants when exposed to cold stress in barley. It was suggested that the interplay of melatonin and ABA helps the plants to maintain better water status. The study of Xing et al. (2019) demonstrated that the improvement in drought tolerance was due to lipoxygenase-mediated elevation in ABA content. On the contrary, melatonin treatment lowered the ABA content thus contributing to improved stomatal performance (Li et al., 2015), germination (Li et al., 2021) and drought tolerance (Ahmad et al., 2022).

Despite several reports demonstrating the potential of melatonin as a phyto-protectant against drought stress in a range of crops and the regulatory mechanisms by which it induces drought tolerance, limited information is available on its role in *Arachis hypogaea* L., where drought stress greatly affects its productivity. More research is also needed to shed light on how melatonin regulates LOX expression and phytohormonal changes during induction of drought tolerance in plants. Therefore, the current work is focused on assessing the mechanistic effects of melatonin priming on antioxidant systems, photosynthetic rate and lipoxygenase expression including JA and ABA levels in drought-distinguished varieties of groundnut.

## 2 Materials and methods

### 2.1 Plant materials and seed priming

Groundnut (*Arachis hypogaea* L.) seeds of two varieties *viz*. Kadiri-7 (K-7; drought-sensitive) and Kadiri-9 (K-9; drought-tolerant) were procured from Agricultural Research Station Kadiri, Anantapur, Andhra Pradesh, India. The pods were sun-dried for 4 h followed by a brief surface sterilization with 70% (v/v) ethanol for 3 min and then washed with autoclaved double distilled water thrice. The seeds were then imbibed with the working solutions of melatonin (Catalog No. M5250, Sigma Aldrich, U.S.A.) of varying concentrations (5 µM, 10 µM, 25 µM, 50 µM and 100 µM) and kept in dark for 24 h. A batch of hydro-primed seeds served as controls. Seeds were dried overnight at room temperature in the dark for back-drying. The overnight dried seeds were evenly placed on moist sterile germination paper to germinate for 4 days.

### 2.2 Growth conditions

The germinated seedlings of equal length were placed in 15 ml tubes filled with sterilized Hoagland’s nutrient (0.5X) solution. The tubes were covered with parafilm and a small perforation was made to allow the seedlings to stand upright, while the radicle was submerged into the media. The seedlings were allowed to grow in culture room at 25 ± 2°C temperature, 65 ± 2% relative humidity and illuminated under light provided by white fluorescent tube lights (65 µmol photons m^-2^s^-1^) under 16:8-h light and dark photoperiod. The nutrient solution was replenished every 3 days during the experimental period. Drought stress was induced on phenotypically uniform 20-day-old seedlings (three fully grown leaves and with a fourth emerging leaf) using an autoclaved 10% PEG-6000 (Polyethylene glycol) solution. Plants were grouped into four treatments *viz*., unprimed plants without PEG stress (C), unprimed plants with PEG treatment (S), primed plants without PEG treatment (PC) and primed plants with PEG treatment (PS). The unprimed plants without PEG stress (C) and primed plants without PEG treatment (PC) were raised in Hoagland’s nutrient (0.5X) solution while unprimed plants with PEG treatment (S) and primed plants with PEG treatment (PS) were subjected to drought stress with PEG-6000 for 4 days until the appearance of wilting symptoms. The fully grown third leaves were collected from the seedlings of different treatments and used for various experiments, which have been carried out with three independent batches of plants, with triplicates per treatment in each batch of plants.

### 2.3 Determination of endogenous melatonin content

The melatonin content in leaves of both varieties was determined according to Arnao and Hernández-Ruiz (2009) with minor modifications. The fresh leaves (100 mg) were cut into small pieces and placed into vials containing 1 ml chloroform followed by overnight shaking at 4°C in the dark. The solvent was evaporated at 4°C under N_2_ gas and the remnant was dissolved in 100 µl acetonitrile followed by a 0.2-micron polyvinylidene fluoride (PVDF) membrane filtration. The melatonin content in the two varieties was quantified using the Shimadzu High-Performance Liquid Chromatography (Kyoto, Japan) and a C-18 column (Phenomenex KINETEX 250 mm X 4.6 mm). An excitation wavelength of 280 nm and the isocratic mobile phase consisting of water and acetonitrile (50:50) at a flow rate of 0.2 L/min were used for detection. Pure melatonin (Sigma Aldrich, U.S.A.) was used as a standard.

### 2.4 Measurement of ROS

Histochemical techniques were employed to visualize reactive oxygen species *viz.*, superoxide ion ( ${\text{O}}_{2}^{-}$ ) and H_2_O_2_ in the leaves of control and treated seedlings by nitrobluetetrazolium (NBT) and diaminobenzidine (DAB) staining solution respectively, according to Ramel et al. (2009).

To quantify the superoxide radical content, 100 mg of fresh leaf samples were macerated in 65 mM potassium phosphate buffer (pH 7.8) followed by centrifugation at 5000 rpm for 15 min. The collected supernatant was mixed with 10 mM hydroxylamine hydrochloride and 65 mM phosphate buffer (pH 7.8) in equal ratio and incubated for 20 min at 25°C under dark conditions. Then, 3.0 mM α-naphthylamine and 8.5 mM sulphanilamide were added to the mixture followed by incubation for 20 min. The absorbance was recorded at 530 nm (Elstner and Heupel, 1976).

The content of hydrogen peroxide was quantified following the protocol of Alexieva et al. (2001). Briefly, 100 mg fresh leaf tissue was homogenized in 0.1% trichloroacetic acid and centrifuged at 12,000 rpm for 15 min. The supernatant was mixed with 1 M potassium iodide and 10 mM potassium phosphate buffer (pH 7) in equal ratio and incubated for 1 h under dark conditions. H_2_O_2_ concentration was determined by measuring the absorbance of the mixture at 390 nm and the values were calculated using a standard curve.

### 2.5 Antioxidant enzyme assay

To extract crude enzyme for antioxidant enzyme assays, 100-150 mg of the fresh leaf tissue was ground to a fine powder using liquid nitrogen. The tissue was macerated in 3% polyvinylpyrollidone (PVP), 1 mM phenylmethyl sulfonyl flouride (PMSF) and 0.1 mM ethylenediaminetetraacetic acid (EDTA) in ice-cold 50 mM phosphate buffer (pH 7.0). The extracts were centrifuged at 4°C for 30 min at 10000 rpm. The supernatants were used to perform antioxidant enzyme assays (Gogorcena et al., 1997). Superoxide dismutase activity was estimated based on the reduction of nitroblue tetrazolium by light in the presence or absence of protein by following the methodology of Beauchamp and Fridovich (1971). Catalase activity was detected based on the catalytic breakdown of H_2_O_2_ according to Patterson et al. (1984). Ascorbate peroxidase activity was estimated by the amount of ascorbate (AsA) oxidized using the protocol devised by Nakano and Asada (1981). Peroxidase activity was estimated according to the method of Lin and Kao (1999).

### 2.6 Measurement of malondialdehyde content and electrolytic leakage

The MDA content was estimated as described by Heath and Packer (1968). Briefly, fresh leaf tissues were homogenized by adding 0.1% (w/v) trichloroacetic acid (TCA) followed by centrifugation at 15,000 *g* at 4°C for 10 min. The supernatants were collected, mixed with 0.5% thiobarbituric acid, diluted in 20% TCA in a ratio of 1:3 and incubated in the water bath at 95°C for 30 min. The reaction was terminated by incubating on ice. The absorbance was measured at 532 and 600 nm. OD_600_ value was subtracted from OD_532_ nm and MDA content was determined using the Lambert-Beer law with an extinction coefficient (ϵM) = 155 mM^-1^ cm^-1^.

Electrolyte leakage was estimated according to Dionisio-Sese and Tobita (1998) by immersing leaf discs in deionized water in a test tube and heated for 2 h at 32°C and the conductivity was measured (EC_a_). Subsequently, the leaf-containing tubes were heated at 120°C for 15 min followed by cooling at room temperature. The conductivity was measured (ECb) and electrolyte leakage was calculated using the formula as described below:

Electrolyte Leakage (%) = (EC_a_/EC_b_) × 100.

### 2.7 Measurement of proline content

Proline content was determined according to Bates et al. (1973). Briefly, 200 mg of leaf samples were ground using liquid nitrogen and homogenized in 3% sulfosalicylic acid followed by centrifugation at 12,000 *g* for 10 min. One ml of homogenate was taken and mixed with 1 ml of acid-ninhydrin and 1 ml of glacial acetic acid in a test tube for 1 h in a water bath at 100°C and then kept on ice to terminate the reaction. Then, 2 ml toluene was added to the reaction mixture with vigorous mixing and left at room temperature for 30 min until the separation of the two phases. The optical density of the chromophore-containing toluene (upper phase) was measured spectrophotometrically at 520 nm. The proline concentration was determined from a standard curve using D-Proline (Sigma-Aldrich, U.S.A.).

### 2.8 Assessment of photosynthetic parameters

#### 2.8.1 Determination of chlorophyll-a fluorescence parameters and chlorophyll content

The concentration of chlorophyll-a was estimated in fully expanded third leaves using the DUAL PAM-100 (Waltz, Germany). The yield of photosystem II (Y(II)), electron transport rate (ETR), photochemical quenching (qP), non-photochemical quenching (NPQ), regulated heat dissipation Y(NPQ) and non-regulated dissipation Y(NO) were estimated at different photosynthetically active radiation (PAR) values. Prior to the measurement of chlorophyll-a fluorescence parameters, the leaf was dark adapted for 15-20 min. The chlorophyll content was estimated according to Lichtenthaler and Wellburn (1983) with some modifications. The leaf discs from each sample were placed in 80% acetone for efficient leaching of pigment followed by its spectrophotometric analysis at 663 nm and 645 nm.

#### 2.8.2 Activity assay of chlorophyll degrading enzymes

The partially extracted chlorophyll was used to determine the activity of the chlorophyll degrading enzymes as per the protocol of Iriyama et al. (1974). The chlorophyll degrading peroxidase activity was estimated at 668 nm according to Aiamla-or et al. (2010) with minor modifications. Chlorophyllase activity was detected at 665 nm using the methodology described by Fang et al. (1998). Pheophytinase was determined spectrophotometrically at 667 nm according to Kaewsuksaeng et al. (2011) with minor modifications.

### 2.9 Determination of lipoxygenase activity

LOX activity was assayed according to Surrey (1964) with some modifications. To partially purify LOX, the crude protein extract was precipitated using 45% ammonium sulfate and then the assays were performed. The reaction mixture consisting of 20 mM borate buffer (pH-6.0), 0.25% linoleic acid, 0.25% tween-20 and 100 µg of partially purified enzyme extract in a total volume of 1.5 ml was incubated at 25°C for 5 min and the reaction was terminated by the addition of 2 ml of absolute alcohol. The cocktail was centrifuged and the absorbance of the supernatant was measured at 234 nm.

For in-gel activity staining, native gel electrophoresis was carried out according to the procedure described by Aanangi et al. (2016) with minor modifications. The partially purified protein (100 µg) was loaded into the wells of the gel and was allowed to run at 4°C. The gel was then rinsed with 100 mM phosphate buffer (pH - 6.8) and incubated in 250 µM linoleic acid solution. The reaction was carried out for 45 min at room temperature. The gel was later rinsed with the phosphate buffer followed by incubation with 0.05% o-dianisidine dihydrochloride solution at room temperature. Then the gel was visualized and photographed under a white-light illuminator.

### 2.10 Quantification of JA and ABA

The JA and ABA levels in the leaves were estimated using Liquid Chromatography-Mass Spectrometry/Mass-Spectrometry (LC-MS/MS) analysis. About 50 mg leaf tissue was homogenized in 500 µl solution of propanol, water and conc. HCl (2: 1: 0.002 vol/vol). The samples were vigorously shaken on a shaker at 4°C for 30 min. One ml dichloromethane was added to each sample followed by shaking for 30 min at 4°C. The mix was then centrifuged at 10000 rpm at 4°C. The lower phase was filtered and used for quantitative analysis of JA and ABA (Pan et al., 2010). The analysis was performed in Agilent Q-TOF LC/MS 6520 series system (Agilent Technologies, U.S.A.) with ZORBAX RX-C_18_ column (4.6×150 mm, 5 µm, Agilent) at 24°C. The mobile phase used was water with 0.1% formic acid (solution A) and methanol with 0.1% formic acid (solution B) on a gradient elution mode with 2 µl injection volume and 0.4 ml/min flow rate. The gradient elution program used was: 1% B at 0 min to 11 min, 40% B up to next 2 min, 70% B for next 2 min, 99% B for next 1 min and then again 1% B for another 4 min. The detected mass range was 100-2000 m/z.

### 2.11 RNA isolation, cDNA synthesis, and quantitative real-time PCR

Total RNA was extracted from different samples of two varieties by CTAB-ammonium acetate method of Zhao et al. (2012). RNA integrity was analyzed by gel electrophoresis and Nanodrop-2000 UV-vis spectrophotometer (Thermo Fisher Scientific, U.S.A.). The OD 260/280 readings were obtained by spectrophotometry to assess the purity and concentration. Two microgram RNA was converted into cDNA using PrimeScript 1^st^ strand synthesis kit (Takara Bio Inc., Japan) following the manufacturer’s protocol. The real-time PCR (Polymerase Chain Reaction) was carried out on Mastercycler Realplex (Eppendorf, Germany) following the thermocycler condition of 95°C for 2 min (initial denaturation), 40 cycles of amplification (95°C for 15 sec, annealing temperature for 20 sec and 72°C for 30 sec). The reaction was terminated and the melting curve was analyzed to confirm whether the amplicon product is of a single reaction. *Actin4*, a housekeeping gene, was used as an internal control for normalization purposes and the relative fold-change in expression was estimated using the 2^-ΔΔCT^ method as described by Livak and Schmittgen (2001).

### 2.12 Protein extraction and quantification of LOX by immunoblot

About 100 mg frozen leaf tissue was homogenized in an extraction buffer containing 50 mM Tris–HCl pH 8.0, 150 mM sodium chloride, 1 mM phenylmethylsulfonyl fluoride, and 10 mM iodoacetamide with protease inhibitor cocktail (Chung et al., 2009). The mix was centrifuged at 12000 rpm at 4°C for 30 min. Two hundred microgram of protein was loaded into each well of 12% polyacrylamide gel and then the gel was blotted onto the nitrocellulose membrane. The membrane was probed with a primary antibody of lipoxygenase (AS06 128, Agrisera, Sweden) with the dilution of 1:750. For equal loading control, anti-histone-H3 (AS10 710, Agrisera, Sweden) was used as primary antibody in the dilution of 1:2000. HRP-conjugated anti-rabbit antibody was used as a secondary antibody.

### 2.13 Statistical analysis

The data obtained are mean values of three independent experiments with three replicates per treatment in each experiment, which were subjected to one-way analysis of variance (ANOVA). The error bars shown in the graphs depict the standard deviation of mean values. One-way ANOVA was performed for the results using the software SigmaPlot (12.0 version). The significance of differences between treatments was computed using Duncan’s multiple range test (DMRT) (P< 0.05).

## 3 Results

### 3.1 Effect of melatonin on drought stress tolerance in sensitive and tolerant varieties

**Figure 1:**
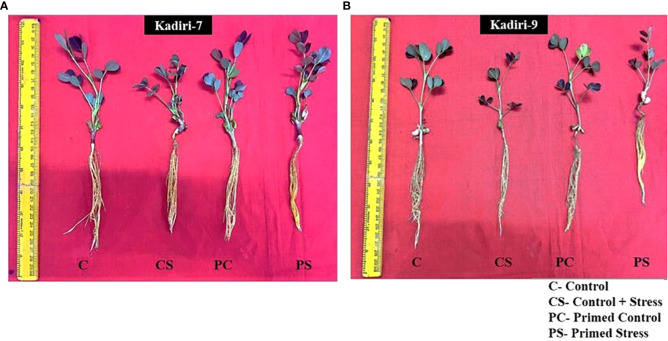
Phenotypic changes of melatonin-primed and unprimed plants of drought-sensitive (K-7) and drought-tolerant (K-9) varieties of groundnut (*Arachis hypogaea* L.) under optimal conditions and PEG-6000 (10%) induced drought stress. C- Plants grown under optimal conditions in Hoagland’s nutrient (0.5X) solution (control); CS- Plants grown by adding PEG in Hoagland’s nutrient (0.5X) solution for 4 days (drought-stressed); PC- Melatonin primed plants grown under optimal conditions in Hoagland’s nutrient (0.5X) solution (primed control); PS - Melatonin primed plants grown by adding 10% PEG-6000 in Hoagland’s nutrient (0.5X) solution for 4 days (primed stress). **(A)** Drought-sensitive variety, Kadiri-7 (K-7); **(B)** Drought-tolerant variety, Kadiri-9 (K-9).

The sensitive variety of groundnut showed wilting whereas the tolerant variety remained healthy under PEG-induced drought stress for 4 days. The effectiveness of melatonin in improving the drought tolerance was examined in Kadiri-7 (K-7) and Kadiri-9 (K-9) varieties of groundnut that differ in their tolerance levels. Of the different concentrations of melatonin used for seed priming, 100 µM was effective in improving the plant growth in the sensitive variety (K-7) under drought stress (**Figure 1A**). The plant height, leaf area and root length of the sensitive variety increased in 100 µM melatonin primed plants as compared to other concentrations tested under drought stress conditions (**Supplementary Figure 1**). Melatonin at higher concentrations (125 and 150 µM) led to a significant decrease in plant height and leaf area while root length remained unchanged in primed stressed plants as compared to unprimed stressed plants in K-7 variety. Surprisingly, there was no significant effect of melatonin (5-100 µM) on the phenotype of the tolerant variety, K-9, under control and stressed conditions (**Figure 1B**; **Supplementary Figure 1**). Thus, exogenous melatonin priming had a distinct effect in ameliorating drought stress tolerance in the sensitive variety while no add-an effect was observed in tolerant variety after 4 days exposure to drought stress.

### 3.2 Endogenous melatonin content in melatonin primed and unprimed stressed plants

**Figure 2:**
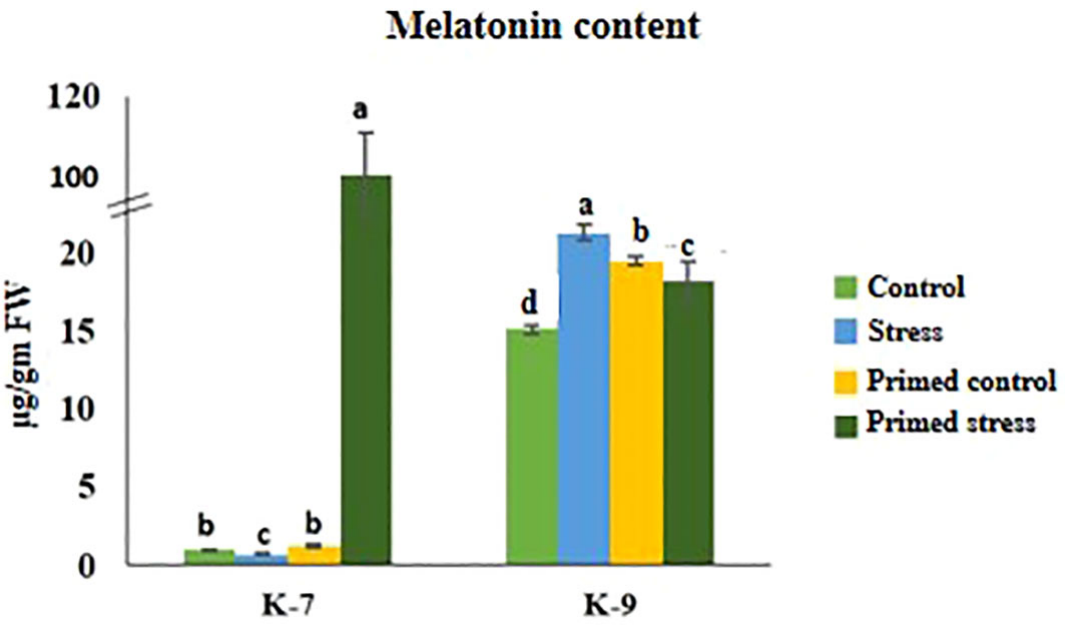
Effect of melatonin priming on endogenous melatonin content in drought-sensitive (K-7) and drought-tolerant (K-9) varieties with or without drought stress. Data represents mean values ± SD of minimum 3 independent experiments. Different alphabets within the group represent significant differences among treatments according to Duncan’s multiple range test at P < 0.05.

Drought stress caused a decrease in melatonin content by 27.3% in K-7. Exogenous melatonin priming caused a marked increase in endogenous melatonin content in K-7 under stress as compared to unprimed stressed plants. However, unprimed plants of K-9 showed 41.16% increased endogenous melatonin content under drought stress compared to its control whereas priming decreased the content by 14.8% compared to unprimed stressed plants (**Figure 2**).

### 3.3 Effect of melatonin on ROS accumulation under drought stress

**Figure 3:**
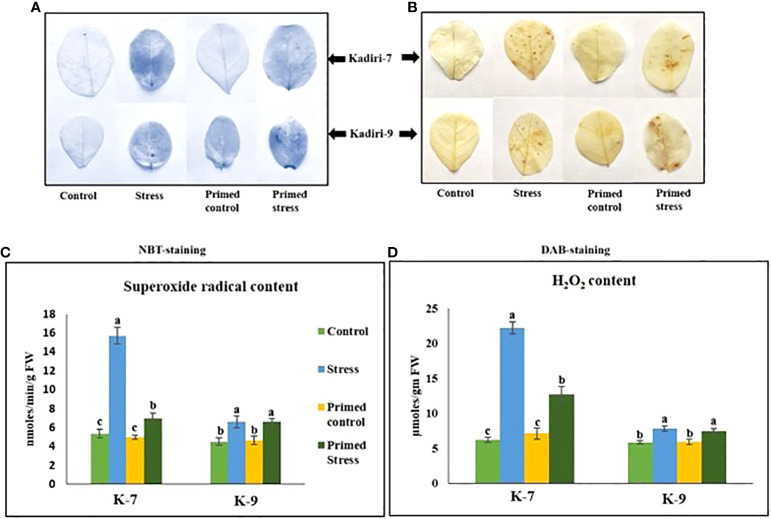
Effects of melatonin on the accumulation of superoxide radicals and hydrogen peroxide in the leaves of drought-sensitive (K-7) and drought-tolerant (K-9) varieties after different treatments. **(A)** Visualization of superoxide radicals in the leaves of different treatments by NBT-staining in K-7 and K-9 varieties; **(B)** H_2_O_2_ accumulation detected by DAB staining in the leaves of K-7 and K-9 varieties after different treatments; **(C)** superoxide radical content and **(D)** H_2_O_2_ content in the leaves of K-7 and K-9 varieties under stress condition. Data represents mean values ± SD of 3 independent experiments. Different alphabets within the group represent significant differences among treatments according to Duncan’s multiple range test at P < 0.05.

Reactive oxygen species like superoxide anion ( ${\text{O}}_{2}^{-}$ ) and hydrogen peroxide (H_2_O_2_) are markers of stress induction. NBT and DAB staining showed higher accumulation of ${\text{O}}_{2}^{-}$ (**Figure 3A**) and H_2_O_2_ (**Figure 3B**) respectively, in the unprimed plants during drought stress in the sensitive variety. However, melatonin priming reduced the accumulation of ${\text{O}}_{2}^{-}$ and H_2_O_2_ under drought. In contrast, the tolerant variety did not show any visible differences in the ROS levels in primed as well as unprimed plants under stress. The content of superoxide radicals (**Figure 3C**) and hydrogen peroxide (**Figure 3D**) significantly increased in both unprimed K-7 and K-9 under stress. However, the ROS levels were comparatively higher in drought stressed plants of K-7 compared to K-9. Melatonin priming considerably decreased the levels of both the ROS molecules under drought stress compared to unprimed plants in K-7, whereas no significant change was observed in K-9 variety.

### 3.4 Effect of melatonin on ROS detoxification

**Figure 4:**
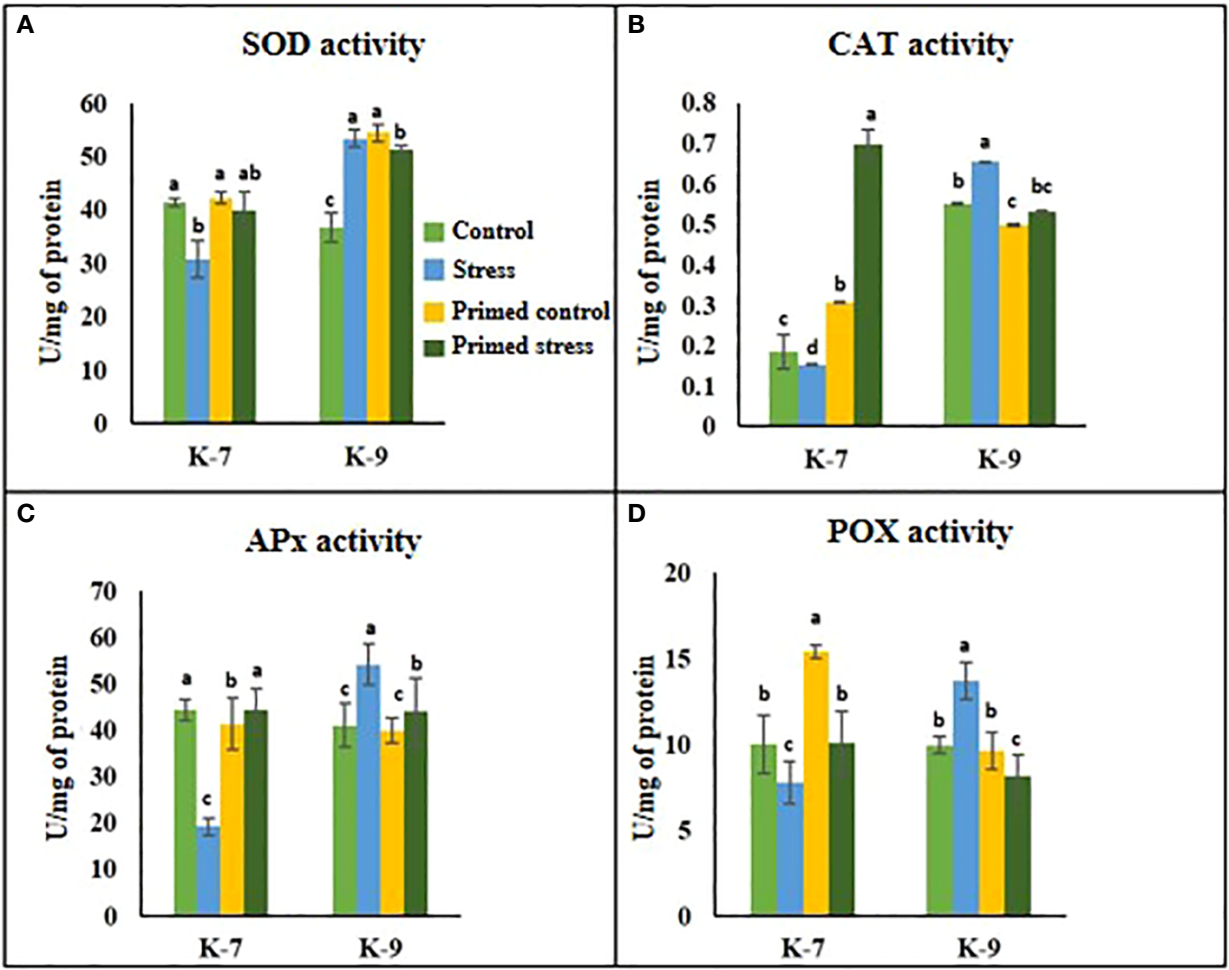
Effects of melatonin priming on antioxidant enzyme activities in drought-sensitive (K-7) and drought-tolerant (K-9) varieties with and without drought stress. **(A)** SOD activity; **(B)** CAT activity; **(C)** APx activity and **(D)** POX activity. Data represents mean values ± SD of 3 independent experiments. Different alphabets within the group represent significant differences among treatments according to Duncan’s multiple range test at P < 0.05.

The role of melatonin on ROS detoxification was investigated by performing several antioxidant enzyme activity assays *viz*., superoxide dismutase (SOD), catalase (CAT), ascorbate peroxidase (APx) and peroxidase (POX). SOD activity decreased by almost 25% in the unprimed plants during drought stress conditions as compared to control plants in K-7 variety. However, its activity increased by 26% in primed plants in comparison to unprimed ones under stress conditions. On the contrary, in the tolerant variety K-9, SOD activity increased by 34.8% in the unprimed plants during stress while it decreased in primed stressed as compared to unprimed stressed plants (**Figure 4A**). The enzyme activities of catalase, ascorbate peroxidase and guaiacol peroxidase declined significantly under stress conditions in comparison to control plants of K-7. Nevertheless, priming significantly increased their activities under stress conditions, thereby allowing the plants to combat drought stress more efficiently. However, in the tolerant variety, CAT, APx and POX activities were comparatively higher but surprisingly priming decreased the activities of these enzymes compared to their respective unprimed plants under stress (**Figures 4B–D**).

### 3.5 Measurement of malondialdehyde content and electrolytic leakage

**Figure 5:**
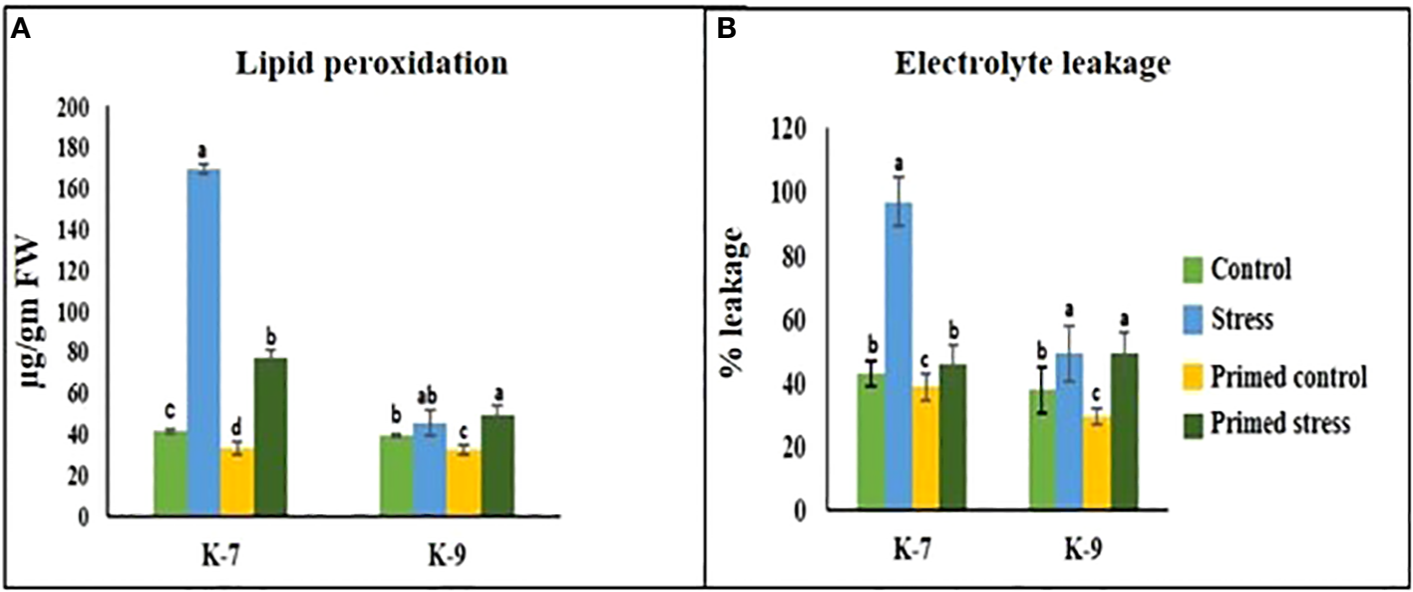
Effect of melatonin priming on **(A)** lipid peroxidation and **(B)** electrolytic leakage in drought-sensitive (K-7) and drought-tolerant (K-9) varieties with or without drought stress. Data represents mean values ± SD of minimum 3 independent experiments. Different alphabets within the group represent significant differences among treatments according to Duncan’s multiple range test at P < 0.05.

A marked increase in MDA content was observed under drought stress in the unprimed plants of K-7. However, the content decreased significantly in primed plants as compared to unprimed plants under stress. Interestingly, the tolerant variety exhibited no notable change in MDA content in unprimed and primed plants under drought stress (**Figure 5A**).

Electrolytic leakage, a hallmark of stress response, is an indicator of membrane integrity. The sensitive variety showed a 125.76% increase in the unprimed plants during stress. However, priming was found to decrease the leakage levels by 52.4% as compared to unprimed plants during stress treatment. In the tolerant variety, there was no statistical difference in electrolytic leakage percentage in unprimed and primed stressed plants (**Figure 5B**).

### 3.6 Proline content

**Figure 6:**
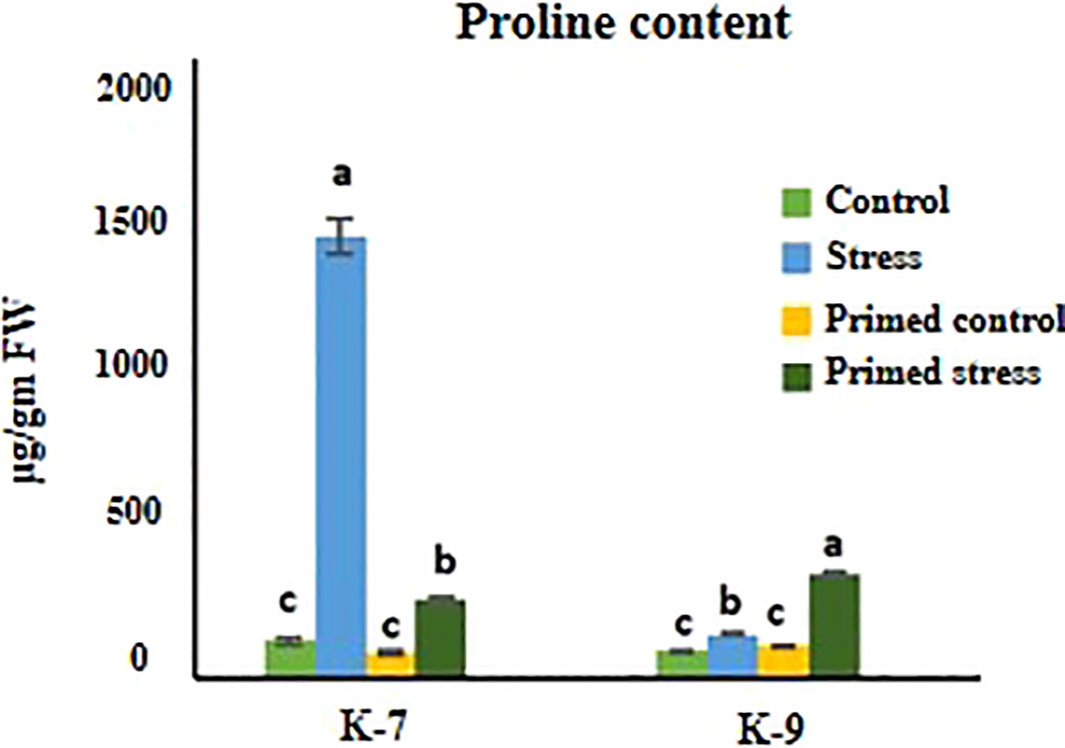
Effect of melatonin priming on proline content in drought-sensitive (K-7) and drought-tolerant (K-9) varieties with or without drought stress. Data represents mean values ± SD of minimum 3 independent experiments. Different alphabets within the group represent significant differences among treatments according to Duncan’s multiple range test at P < 0.05.

Exposure to drought stress caused a pronounced increase in proline content in unprimed plants as compared to controls in K-7. However, the proline content decreased considerably in primed plants as compared to unprimed plants under drought stress. On the other hand, the primed plants exhibited higher proline content than unprimed plants under stress in K-9 variety (**Figure 6**).

### 3.7 Assessment of photosynthetic parameters (Chlorophyll a fluorescence, chlorophyll content and chlorophyll degrading enzyme activities)

**Figure 7:**
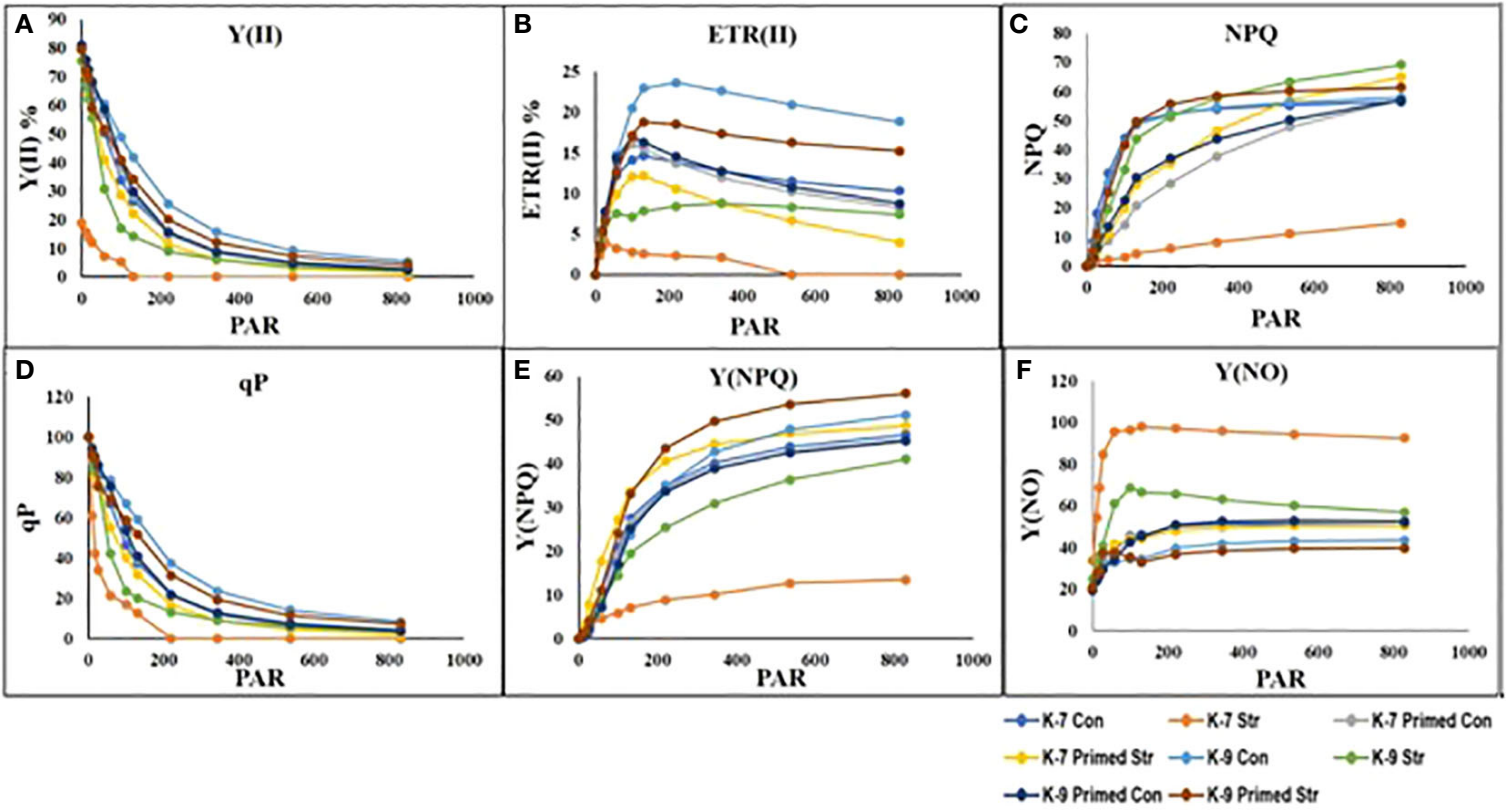
Changes in rapid light curves of photosynthetic parameters **(A-F)** in melatonin-primed or unprimed drought-sensitive (K-7) and drought-tolerant (K-9) varieties of groundnut in optimal conditions (control) or after exposure to drought stress. **(A)** Effective photochemical quantum yield [Y (II)]; **(B)** Electron transport rate of PSII [ETR(II)]; **(C)** Non-photochemical quenching (NPQ); **(D)** Photochemical quenching (qP); **(E)** Regulated heat dissipation Y(NPQ); and **(F)** Non-regulated heat dissipation [Y(NO)]. PAR is photosynthetically active radiation.

**Figure 8:**
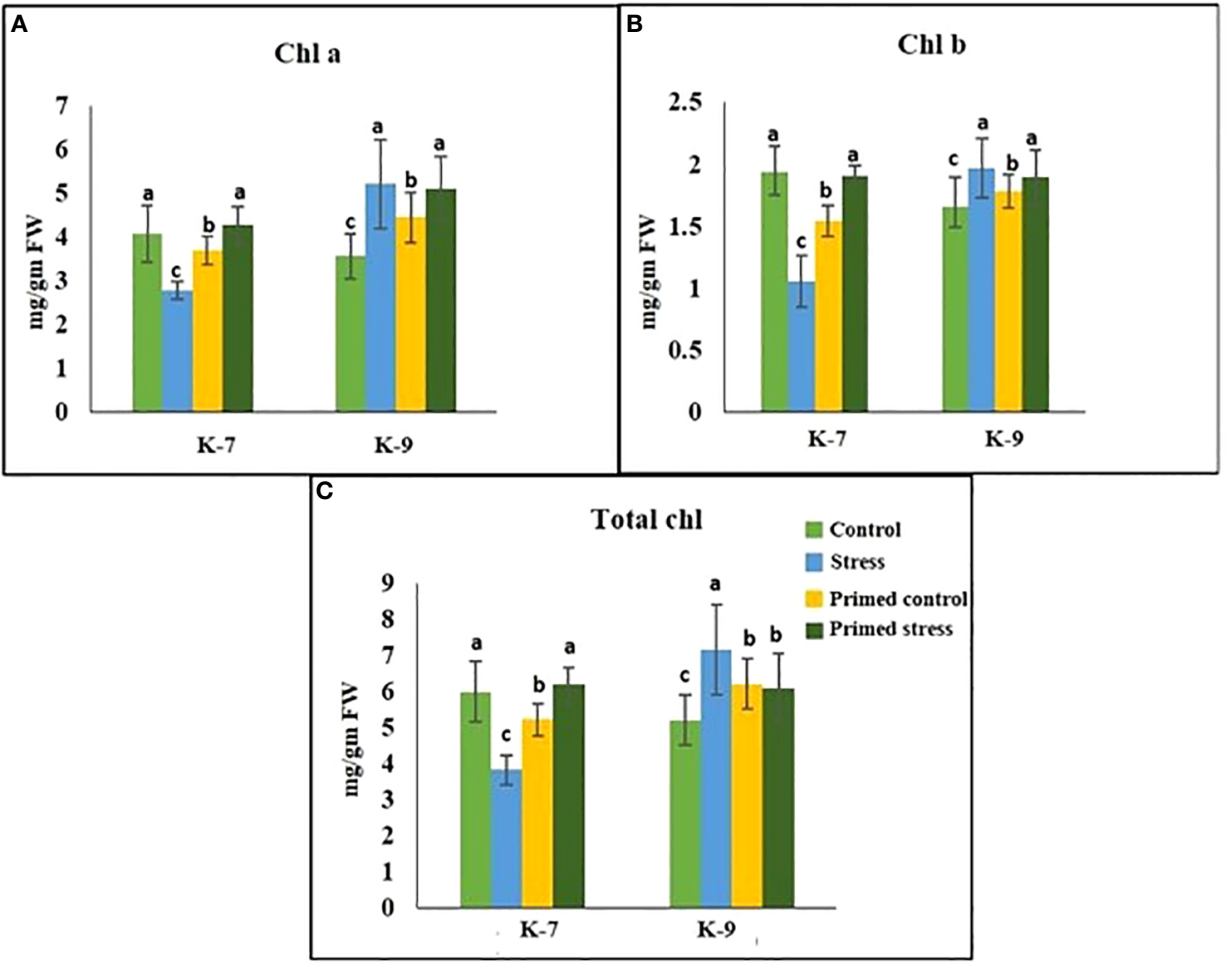
Effect of melatonin priming on **(A)** Chl a; **(B)** Chl b; and **(C)** total chlorophyll content in drought-sensitive (K-7) and drought-tolerant varieties (K-9) with or without drought stress. Data represents mean values ± SD of minimum 3 independent experiments. Different alphabets within the group represent significant differences among treatments according to Duncan’s multiple range test at P < 0.05.

**Figure 9:**
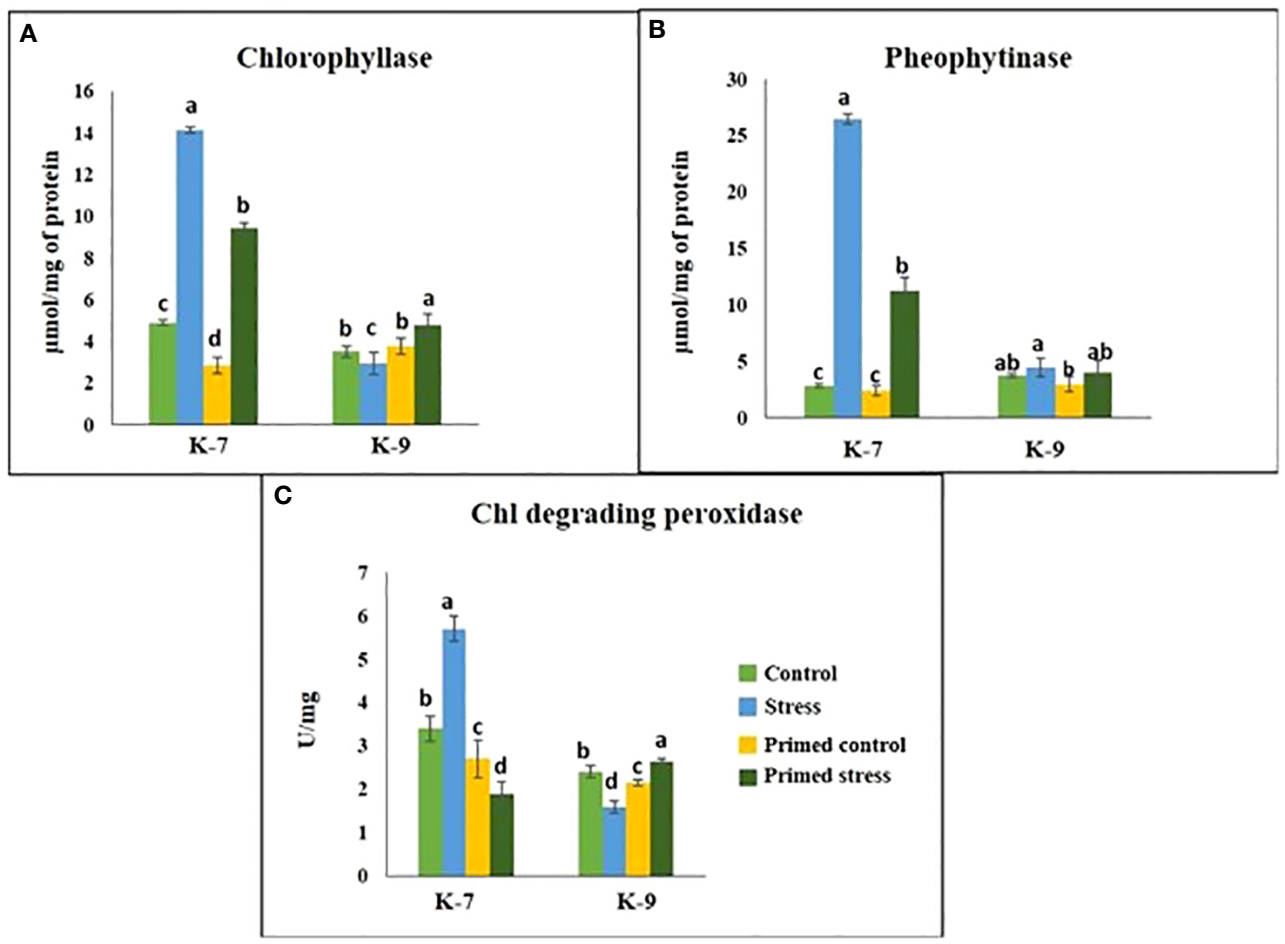
Effect of melatonin priming on the activities of chlorophyll degrading enzymes in drought-sensitive (K-7) and drought-tolerant (K-9) varieties under drought stress. **(A)** Chlorophyllase; **(B)** Pheophytinase; and **(C)** Chlorophyll degrading peroxidase. Data represents mean values ± SD of minimum 3 independent experiments. Different alphabets within the group represent significant differences among treatments according to Duncan’s multiple range test at P < 0.05.

The chlorophyll-a fluorescence characteristic exhibited marked differences between the two varieties under stress conditions. Stress condition dramatically decreased the photosystem II yield Y(II), which reached zero from ≥131 PAR in unprimed plants of K-7 variety. However, the level was found to be similar under all PAR values during stress compared to controls in unprimed plants of K-9 variety. Priming elevated Y(II) values significantly in primed stressed plants compared to unprimed stressed in both varieties although K-7 showed more prominent differences (**Figure 7A**). Drought stress had a distinct effect on photosystem II electron transport rate (ETR) in K-7 variety where the values decreased significantly from ≥27 PAR and declined to zero from ≥536 PAR onwards. K-9 unprimed stressed plants also showed a decrease in ETR (II) values after ≥58 PAR compared to unprimed control plants. Primed stressed plants exhibited higher ETR (II) compared to unprimed stressed in both varieties (**Figure 7B**). Non-photochemical quenching (NPQ), photochemical quenching (qP) and regulated heat dissipation Y(NPQ) levels were considerably decreased in K-7 plants during stress. However, K-9 unprimed stressed plants showed no marked changes in NPQ and Y(NPQ) levels compared to control plants under stress. Melatonin ameliorated both parameters in primed stressed plants compared to unprimed stressed plants of the sensitive variety. In the case of K-9, priming did not exhibit any beneficial effect (**Figures 7C–E**). The level of non-regulated heat dissipation Y(NO) increased notably in unprimed stressed plants of K-7 compared to control ones. Priming mitigated Y(NO) level under stress compared to unprimed stressed plants. K-9 variety also showed a significant increase in Y(NO) level in unprimed stressed plants from ≥58 PAR compared to control plants. The Y(NO) of K-9 primed stressed plants exhibited no significant change compared to unprimed stressed plants (**Figure 7F**). The chlorophyll a and b contents decreased by 31.8% and 45.6% under stress in K-7, respectively. Both the contents increased significantly in primed plants under stress conditions. K-9 variety showed 46.2% and 18.8% higher chlorophyll a and b contents, respectively under stress compared to controls. Surprisingly, there was no significant change in chlorophyll a and b contents in primed stressed plants compared to unprimed stressed plants of K-9 (**Figures 8A, B**). The total chlorophyll content of unprimed K-7 showed a significant decrease compared to control under stress. But priming caused a notable increase in the total chlorophyll content under stress conditions. The tolerant K-9 variety was found to have significantly higher total chlorophyll content compared to control but surprisingly priming decreased the content compared to unprimed plants during stress (**Figure 8C**). Drought stress stimulates the chlorophyll degradation process, which is detrimental to plant survival. The chlorophyllase (**Figure 9A**), pheophytinase (**Figure 9B**) and chlorophyll degrading peroxidase (**Figure 9C**) activities increased significantly in unprimed stressed plants, respectively compared to controls in K-7 variety. Priming reduced the chlorophyll degradation as primed stressed plants showed 27.5%, 57.5% and 66.7% decrease in the activities of these enzymes compared to unprimed stressed plants of K-7, respectively. Drought stress decreased the chlorophyllase and chlorophyll degrading peroxidase activities significantly by 16.0% and 33.3% whereas pheophytinase activity did not show any significant change in unprimed plants of K-9. Surprisingly, chlorophyll degrading peroxidase and chlorophyllase activities increased by 65% and 62.7% while pheophytinase activity was similar in primed stressed plants compared to unprimed stressed plants.

### 3.8 Determination of lipoxygenase activity

**Figure 10:**
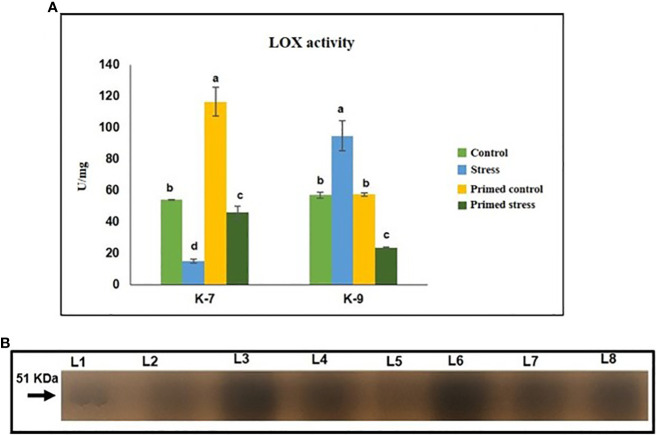
Effect of melatonin priming on the activity of lipoxygenase enzyme in drought sensitive (K-7) and drought-tolerant (K-9) varieties under drought stress. **(A)** Lipoxygenase activity (spectrophotometric detection) and; **(B)** in-gel staining activity. Data represents mean values ± SD of minimum 3 independent experiments. Different alphabets within the group represent significant differences among treatments according to Duncan’s multiple range test at P < 0.05.

Lipoxygenases are a family of enzymes that are reported to have a protective role against abiotic and biotic stresses. Drought stress significantly decreased LOX activity when compared to the control plants in the sensitive variety. Melatonin priming caused a marked increase in its activity in K-7 compared to unprimed stressed plants. On the contrary, drought stressed plants of K-9 variety exhibited higher activity than that of controls. However, primed stressed plants showed significantly decreased LOX activity as compared to unprimed stressed plants in K-9 (**Figure 10A**). The results of in-gel activity also correlated with the LOX activity assay (**Figure 10B**).

### 3.9 JA and ABA quantification

**Figure 11:**
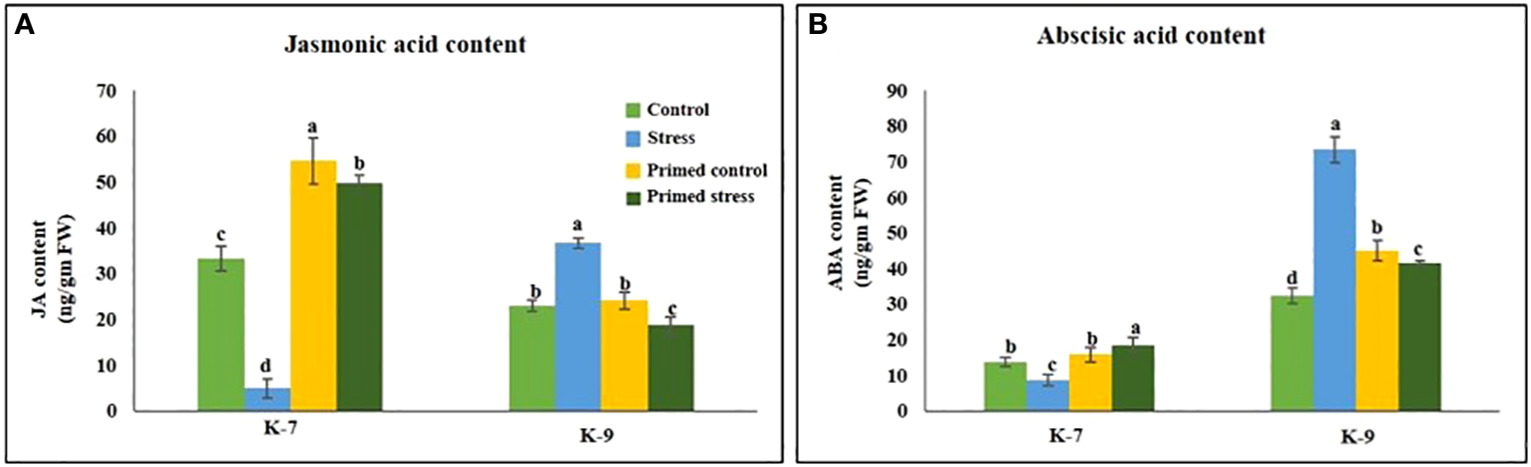
Effect of melatonin priming on the contents of **(A)** jasmonic acid and **(B)** abscisic acid in drought-sensitive (K-7) and drought-tolerant (K-9) varieties under drought stress. Data represents mean values ± SD of minimum 3 independent experiments. Different alphabets within the group represent significant differences among treatments according to Duncan’s multiple range test at P < 0.05.

Drought stress significantly reduced the level of JA level as compared to control plants in sensitive variety. However, melatonin priming increased its content by 10.1-fold under stress. Contrarily, the tolerant variety exhibited higher JA levels in unprimed plants under stress but surprisingly, priming led to its decrease under stress conditions (**Figure 11A**).

The ABA content in sensitive variety was lower as compared to the tolerant variety. Drought stress significantly decreased the ABA content whereas priming enhanced the content by 2.1-fold in the sensitive variety. The tolerant variety exhibited higher ABA content under stress whereas priming decreased it by 1.8-fold (**Figure 11B**).

### 3.10 Relative expression of genes related to drought stress

**Figure 12:**
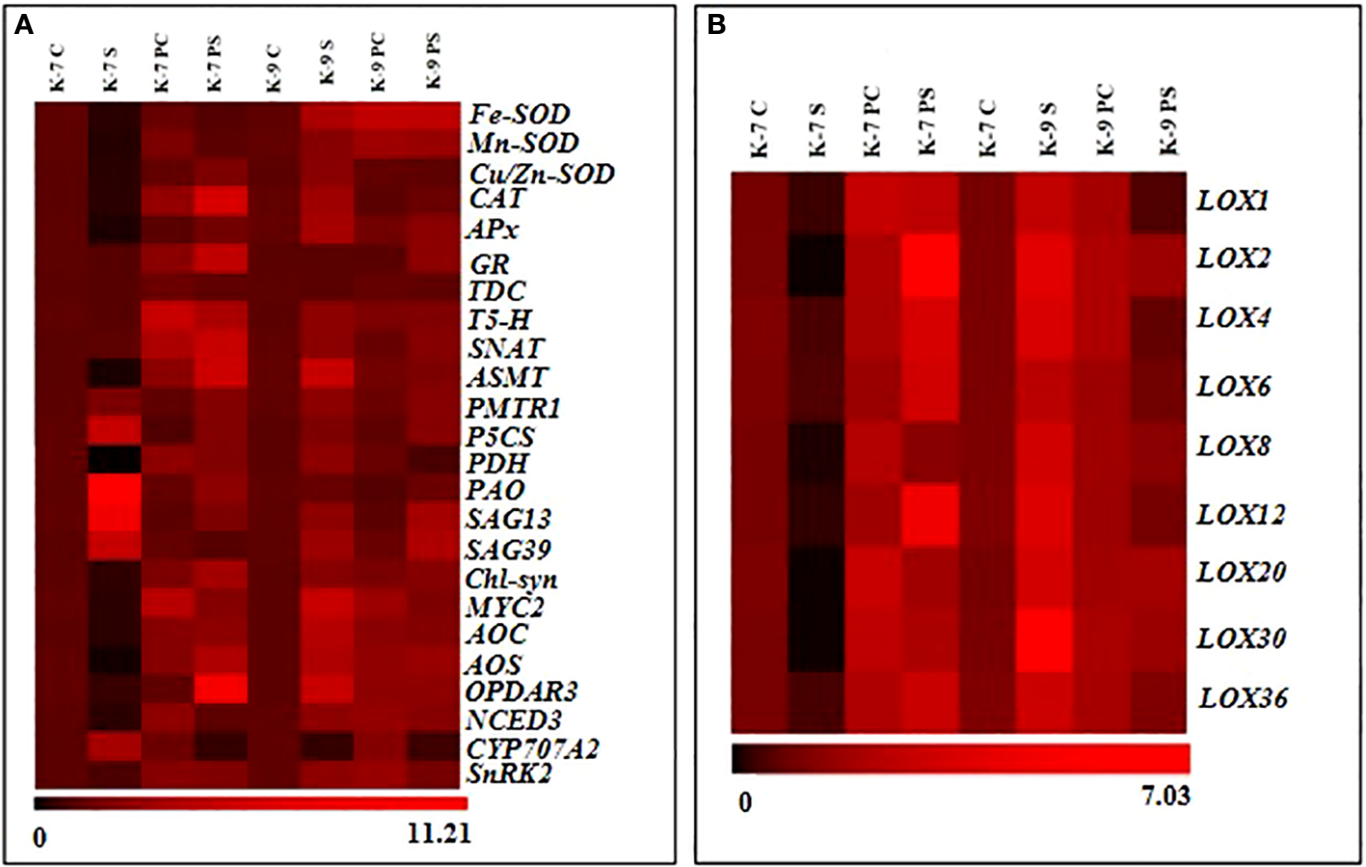
Relative abundance of mRNA transcripts of genes (fold-change in comparison to control plants of respective variety) associated with **(A)** antioxidant, melatonin biosynthesis and receptor, chlorophyll degradation and biosynthesis, JA biosynthesis, ABA biosynthesis and degradation, and ABA response element **(B)** lipoxygenase isoforms in primed or unprimed plants K-7 (drought-sensitive) and K-9 (drought-tolerant) varieties, under optimal conditions (control) or drought stress.

The expression level of the genes involved in stress modulation *viz*., ROS regulation (Fe-SOD, Mn-SOD, Zn-SOD, CAT, APx and GR); melatonin biosynthesis (TDC, T-5H, SNAT and ASMT); melatonin receptor (PMTR1); chlorophyll degrading (PAO, SAG13 and SAG39); chlorophyll synthesizing (Chl-Syn); lipoxygenases and related (LOX1, 2, 4, 6, 8, 12, 20, 30, 36, AOC, AOS, OPDAR3, MYC2); ABA related (NCED3, CYP707A2, SnRK2); proline related (P5CS and PDH) was analyzed to confirm biochemical, physiological and molecular data (**Figure 12A**). Drought stress downregulated the expression of genes of antioxidant enzymes significantly in K-7 compared to controls. Priming was found to have a positive effect as it increased the expression of these genes by 3.0, 4.6, 6.5, 16.2, 7.2 and 4.7-fold compared to unprimed plants under stress, respectively. The tolerant variety, K-9 showed increased expression of these genes under stress in unprimed plants except for the expression of *GR* where there was no significant change in its expression. The expression levels of *Fe-SOD*, *Mn-SOD* and *GR* were elevated, whereas the expression levels of *Zn-SOD*, *CAT* and *APx* decreased significantly in primed stressed plants compared to unprimed stressed plants of K-9 variety (**Supplementary Figures 2A–F**). Exogenous melatonin modulated the expression of melatonin biosynthesis genes under stress conditions. The expression level of *TDC* (Tryptophan decarboxylase), *T5H* (Tryptophan-5 hydroxylase) and *ASMT* (Acetyl seratonin methyl transferase) decreased significantly whereas *SNAT* (Seratonin N-acetyl transferase) showed no significant change in unprimed stressed plants compared to control stressed plants in K-7 (**Supplementary Figures 3A–D**). Priming elevated the expression of *TDC*, *T5H*, *SNAT* and *ASMT* notably under stress compared to unprimed stressed plants of K-7. In the tolerant variety K-9, the expression level of *T5H*, *SNAT* and *ASMT* genes showed an increase under stress in unprimed plants, whereas there was no significant change in the expression of *TDC* level in unprimed stressed plants. Interestingly, primed stressed plants either showed no significant change (*TDC* and *SNAT*) or decreased expression (*T5H* and *ASMT*) compared to unprimed stressed plants (**Supplementary Figures 3A–D**). *PMTR1* (Plant melatonin receptor) exhibited similar expression patterns in both the varieties independent of the treatment (unprimed and primed) and conditions (optimal or drought) (**Supplementary Figure 4**). The expression of chlorophyll degrading genes *PAO* (Pheophorbide A oxygenase), *SAG13* and *SAG39* (Senescence associated genes) increased significantly by 11.0, 7.2 and 4.3-folds in K-7 in unprimed stressed plants compared to controls (**Supplementary Figures 5A–D**). Although stress caused an increase in the expression of *PAO*, *SAG13* and *SAG39* genes in K-9 variety, the levels were lower than the unprimed stressed plants of K-7 variety. Priming downregulated their expression by 5.1, 4.0 and 4.8-fold in K-7 compared to unprimed plants under stress. Interestingly, the expression levels of *SAG13* and *SAG39* genes increased while *PAO* expression remained unchanged compared to unprimed stressed plants in K-9 variety. The chlorophyll synthesis gene (*Chl-Syn*) expression decreased 2.7-fold in stressed plants compared to control and increased 7.7-fold in primed stressed plants compared to unprimed stressed plants of K-7. The expression level of the *Chl-Syn* increased in unprimed and primed stressed plants as compared to controls (**Supplementary Figure 5A–D**). The expression of the proline biosynthesis gene, *P5CS* (Pyrolline-5 carboxylase) increased whereas the expression of the degrading gene, *PDH* (Proline dehydrogenase) decreased significantly in unprimed plants of K-7 exposed to stress. On the other hand, the expression of *P5CS* decreased in primed stressed plants while the expression of *PDH* increased as compared to unprimed stressed plants. *P5CS* and *PDH* gene expressions increased 1.9-fold and 2.2-fold in unprimed stressed plants compared to controls in K-9, respectively. The primed plants showed no change in the expression of *P5CS* whereas *PDH* expression decreased compared to unprimed plants under stress in K-9 (**Supplementary Figures 6A, B**).

The lipoxygenase isoforms (*LOX1, 2*, *4*, *6*, *8*, *12*, *20*, *30* and *36*) exhibited similar expression patterns (**Figure 12B**). K-7 showed a decrease in the transcript levels of these isoforms whereas the transcript levels significantly increased in K-9 under stress as compared to their controls. However, priming enhanced the expression in K-7, whereas in K-9, the levels decreased considerably compared to their respective unprimed plants under stress (**Supplementary Figures 7A–I**). The genes related to jasmonic acid biosynthesis *viz*., *AOC* (Allene oxide cyclase), *AOS* (Allene oxide synthase) and *OPDAR3* (Oxophytodienoic acid reductase 3) decreased significantly by 3.0, 12.6 and 2.3-folds in stressed plants compared to control plants in K-7, respectively (**Supplementary Figures 8A–C**). Melatonin priming significantly increased their expression compared to unprimed plants under stress. However, the expression of these genes increased in K-9 under stress as compared to controls. Interestingly, priming decreased the expression significantly as compared to unprimed plants under stress. The *MYC2* expression level decreased by 2.71-folds in K-7 and increased by 4.3-folds in K-9 stressed plants compared to their respective control plants. Priming under stress showed increased expression of *MYC2* in K-7 by 6.2-fold, but in K-9 the expression decreased by 2.5-folds as compared to unprimed stressed plants (**Supplementary Figure 8D**). The expression of ABA biosynthesis gene, *NCED3* (9-cis-epoxycarotenoid dioxygenase 3) was significantly downregulated under stress when compared to its control in the sensitive variety. Priming enhanced its expression under stress compared to unprimed plants under stress. The tolerant variety showed higher transcript levels of *NCED3* under stress. However, melatonin priming caused a decline in its expression under stress (**Supplementary Figure 9A**). Contrasting expression pattern was observed for the ABA degrading gene *i.e.*, *CYP707A2* (ABA 8’-hydroxylase) where a significant increase in its expression was observed in primed stressed plants of K-7 unlike in K-9 where it decreased under stress. However, its expression in primed stressed plants was lower in K-7 whereas it was found to be higher in K-9 variety in comparison to unprimed stressed plants (**Supplementary Figure 9B**). *SnRK2* (Snf-related protein kinase 2), an important gene in ABA signaling response, showcased a similar expression pattern as that of *NCED3* (**Supplementary Figures 9A–C**).

### 3.11 Immunoblot analysis of LOX

**Figure 13:**
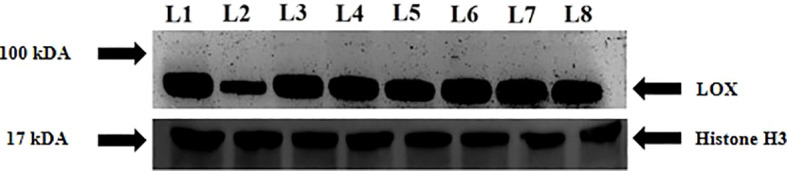
Immunoblot analysis of lipoxygenase in melatonin primed and unprimed plants of drought-sensitive (K-7) and drought-tolerant (K-9) varieties of *Arachis hypogaea* L. under optimal and drought stress conditions. Lane 1- K-7 control, Lane 2- K-7 stress, Lane 3- K-7 primed control, Lane 4- K-7 primed stress, Lane 5- K-9 control, Lane 6- K-9 stress, Lane 7- K-9 primed control and Lane 8- K-9 primed stress.

Immunoblot analysis revealed differences in LOX expression in sensitive and tolerant varieties upon exposure to drought stress. The intensity of the protein decreased under stress in the sensitive variety whereas priming increased the intensity of the protein under stress. In contrast, the intensity of protein increased in K-9 stressed plants as compared to unstressed plants whereas it decreased in primed stressed plants than in primed control plants (**Figure 13**).

## 4 Discussion

Plant growth is a complex biochemical and physiological phenomenon being modulated by several plant growth regulators such as auxin, cytokinin, jasmonic acid, gibberellins, abscisic acid, ethylene and brassinosteroids (Gray, 2004). Seed priming with many of these growth regulators has been proven to alleviate the adverse effects of abiotic as well as biotic stresses. Melatonin priming also has been found to assist plants to endure drought stress by maintaining ROS homeostasis and also by activating stress-related transcription factors and genes. Here, the role of melatonin on redox homeostasis, photosynthesis and lipoxygenase expression to mitigate the negative impacts of drought in *Arachis hypogaea* L. was investigated in K-7 (drought-sensitive) variety in comparison to K-9 (drought-tolerant) of groundnut.

### 4.1 Melatonin alleviates PEG-induced drought stress in the sensitive variety

Melatonin priming exhibited beneficial effects on the morphology of the sensitive variety as evidenced by fewer wilting symptoms with increased plant height, root length and leaf area after exposure to 4-days of drought stress treatment as compared to unprimed stressed plants. However, melatonin-primed and unprimed plants of tolerant variety (K-9) did not show any differences in plant height, root length and leaf area when subjected to the same duration of drought stress treatment. It can be interpreted that exogenous melatonin has the potential to improve the drought tolerance only when severe stress is prevalent as in the sensitive variety after 4 days of exposure to drought stress, unlike the tolerant variety which was inherently equipped to withstand this duration of stress treatment (**Figure 1**; **Supplementary Figure 1**). Similar findings have been reported in cotton where melatonin priming improved the drought tolerance of sensitive variety while no significant phenotypic effect was noticed in the tolerant variety of cotton under drought stress (Supriya et al., 2022).

### 4.2 Exogenous priming increases endogenous melatonin content

Previous studies have shown that melatonin plays a significant role in drought stress tolerance. In this study, the marked increase in melatonin content with upregulated expression of genes involved in melatonin biosynthesis in primed stressed plants of sensitive variety justifies its implication in stress tolerance. This is further evident by an increase in the melatonin content in unprimed stressed plants of the tolerant variety. However, the decrease in melatonin content in tolerant primed stressed plants compared to unprimed stressed plants (**Figure 2**) signify the feedback inhibition, which is consistent with findings of Bhowal et al. (2021). Further, down-regulated expression of *T-5H* and *ASMT* in this variety (**Supplementary Figures 3B, D**) also correlated to the decreased endogenous melatonin content. The unchanged expression of *PMTR1*, which is a receptor of melatonin, indicates that variation in endogenous melatonin was not due to differences in the expression of receptor genes (**Figure 4** and **12A**). This also edicts the ambiguity about the position and/or function of PMTR1, as suggested by Lee and Back (2020).

### 4.3 Priming alleviates oxidative damage and maintains ROS homeostasis

Plants produce more ROS under drought stress conditions, which causes oxidative damage to the cells and tissues (Kar, 2011). Melatonin scavenges these ROS molecules and gets converted into metabolites *viz.*, AFMK (N1-acetyl-N2-formyl-5- methoxyknuramine) and AMK (N1-acetyl-5-methoxykynuramine) that are also involved in several cascades of reactions to scavenge other types of ROS, thereby maintaining the redox homeostasis more effectively (Ressmeyer et al., 2003). The role of melatonin in the mitigation of hydrogen peroxide levels and the augmentation of antioxidant systems has been reported in *Malus* (Li et al., 2015). Here, the higher accumulation of superoxide and hydrogen peroxide radicals in leaves of unprimed plants and their decreased levels in primed plants of K-7 under stress justifies the scavenging property of melatonin (**Figure 3**). On the contrary, melatonin primed stressed plants did not show any notable changes in staining intensity as compared to unprimed stressed plants in the tolerant variety, K-9 indicating that exogenous priming has no further effect on ROS levels.

Plants have developed several enzymatic and non-enzymatic defense systems to combat oxidative damage by minimizing stress-induced ROS accumulation (Qamer et al., 2021). Buttar et al. (2020) reported that exogenous melatonin activates the antioxidant enzymatic activities to reduce stress-induced ROS bursts in wheat seedlings. In our study, decreased antioxidant enzymatic activities in the sensitive variety under stress (**Figures 4A–D**) correlated with the decrease in the transcript levels of the genes like *Cu-SOD*, *Mn-SOD*, *Zn-SOD*, *CAT*, *APx* and *GR* thereby resulting in increased ROS accumulation. Melatonin aided the activation of the antioxidant defense machinery by upregulating the expression of these stress-related genes and their activities to scavenge intracellular ROS (**Figure 12A**; **Supplementary Figures 2A–F**). Thus, melatonin mediated maintenance of cellular redox homeostasis by elevating the antioxidative defense mechanism, thereby improving the drought tolerance of the sensitive variety of groundnut.

### 4.4 Melatonin ensures membrane integrity and stability by decreasing lipid peroxidation and electrolytic leakage

Unregulated intracellular ROS levels under stress condition lead to membrane lipid peroxidation followed by membrane rancidity. Electrolytic leakage is caused by lipid peroxidation, which leads to membrane damage, a repercussion of drought stress (Jaleel et al., 2007). Our results showed that drought stress intensifies the generation of ROS in groundnut, resulting in higher levels of electrolytic leakage and MDA which are crucial oxidative-damage indicators of cell membrane integrity. Similarly, Khalvandi et al. (2021) reported that drought stress caused damage to the membrane system which increased lipid peroxidation and plasma membrane electrolytic leakage in winter wheat. In this research, lipid peroxidation and electrolytic leakage were alleviated in the sensitive variety upon melatonin priming under stress. The reason might be the amphipathic nature of melatonin that tends to spread across the cytoplasm and lipid membranes. Thus, the interfacially positioned melatonin inhibits lipid peroxidation in biological membranes by directly neutralizing hazardous reactants (de Lima et al., 2010). The reduced lipid peroxidation and electrolyte leakage in the unprimed tolerant variety during stress in comparison to the sensitive variety, explains its inherent ability to tolerate the drought stress (**Figures 5A, B**).

### 4.5 Melatonin maintains osmotic potential by regulating proline levels

Plants tend to cope with environmental stresses by regulating the osmotic potential and proline is a crucial osmolyte involved in tolerance to various stresses. Several studies have reported an increase in intracellular proline levels during exposure to stress in different plant species (Liang et al., 2013). Similarly, a significant increase in proline levels was observed under drought stress which is more pronounced in K-7 as compared to K-9 variety in the present study. Besides being an osmoprotectant and cellular stabilizer, it imparted toxic effects if over-accumulated as observed in tomato, where an imbalance in inorganic ions was observed (Heuer, 2003). Similar findings have been reported by Roy et al. (1993) where low concentrations of exogenously applied proline was effective in ameliorating the adverse effects of salinity whereas higher concentrations reduced the seedling growth in rice. Our results showcased higher proline accumulation under stress which might be due to the repression of the proline catabolic gene, *PDH* in mitochondria whose activity might be hampered by the oxidative burst inside the cell in sensitive variety (**Figure 6**). However, the regulated expression of *P5CS* and *PDH* might have contributed to the balanced proline levels in the tolerant variety under stress. Therefore, our findings suggest a vital role of melatonin in proline metabolism in plants by maintaining an equilibrium between the expression of proline biosynthetic (*P5CS*) and proline catabolic (*PDH*) genes (**Figure 12A**; **Supplementary Figures 6A, B**).

### 4.6 Melatonin inhibits chlorophyll degradation and senescence

Chlorophyll-a fluorescence has been commonly used to assess the plant photosynthetic performance under a variety of stress conditions (Govindjee, 2004). Severe drought stress is reported to cause photoinhibition in the PSII reaction center (Sperdouli and Moustakas, 2012). In line with these observations, we observed that Y(II), ETR(II), qP and NPQ were reduced dramatically in the sensitive variety during drought stress. Melatonin has been reported to boost photosynthetic efficiency in higher plants under drought stress in cucumber (Zhang et al., 2013). In this study, melatonin priming augmented the yield of PSII, electron transport rate (ETR) in K-7, similar to the report of Wang et al. (2013) justifying the role of melatonin in maintaining better photosynthetic activities under drought. As priming did not cause any significant change in endogenous melatonin content in K-9, no change in photosynthetic rate was observed. Y(NO) and Y(NPQ) are two important components of the photosynthetic machinery. Higher Y(NPQ) and lower Y(NO) in primed plants of K-7 justify the proper functioning of its xanthophyll-carotenoid cycle, similar to the report of Khan et al. (2019). As such melatonin priming did not cause any changes in Y(NPQ) and Y(NO) as compared to unprimed plants in the tolerant variety, K-9 under stress showing no additional effect of exogenous melatonin on photosynthesis (**Figures 7A–F**).

Plants have to be protected from free radicals and associated oxidative stress since chloroplast is the primary location of free radical production. Chlorophyll degradation leads to senescence in plants which is detrimental to plant growth and development (Hörtensteiner, 2006). Previous studies have shown that exogenous melatonin priming induces endogenous melatonin production (Antoniou et al., 2017). Our findings imply that exogenous application can alter endogenous melatonin accumulation in drought-stressed plants of sensitive variety that can help to retain chloroplast integrity and increase the net photosynthetic rate. This is further supported by increased chlorophyll content upon melatonin priming in the sensitive cultivar under stress (**Figures 8A–C**). An increased expression of the chlorophyll synthesis gene (*Chl-syn*) (**Supplementary Figure 5D**) and decreased activities of chlorophyll degrading enzymes such as chlorophyllase, pheophytinase and chlorophyll degrading peroxidase upon melatonin priming in the sensitive variety emphasizes the role of melatonin against chlorophyll degradation (**Figures 9A–C**). Similarly, Ma et al. (2018) reported that drought-induced leaf senescence suppressed by cytokinin and melatonin, was marked by the down-regulation of chlorophyll-degradation genes and enzyme activities in creeping bentgrass (*Agrostis stolonifera*).

Senescence is a critical measure of chlorophyll degradation. The expression levels of *PAO* and senescence-associated genes (*SAG13, 39*) was found to decrease upon priming in K-7 under stress (**Figure 12A**; **Supplementary Figures 5A–C**). Thus, the activity of these chlorophyll-degrading enzymes in primed stressed plants was alleviated, thereby inhibiting senescence which could be ascribed to the melatonin treatment. Increased endogenous melatonin concentration could be responsible for these substantial changes, which leads to improved photosynthetic machinery. The enhanced photosynthetic efficiency and lowered expression of chlorophyll degrading genes under stress conditions in unprimed plants of tolerant variety are supportive of its inherent drought tolerance capacity (**Figures 9** and **12A**; **Supplementary Figure 5**).

### 4.7 Melatonin enhances JA and ABA biosynthesis by stimulating lipoxygenases under drought stress

LOXs are non-heme iron-containing dioxygenases enzymes that catalyze the conversion of polyunsaturated fatty acids to hydroperoxy fatty acids, which are involved in the formation of stress-related plant growth regulators such as jasmonic acid (JA), methyl jasmonate (MeJA) and so on (Blée, 2002). Despite greater substrate availability (linoleic and linolenic acid) due to a higher rate of lipid peroxidation, decreased activity and transcripts level of LOX under stress in unprimed K-7 suggested its catalytic inhibition by thiol sensitive feedback regulation, as described by Maynard et al. (2021). The elevated LOX activity and content in primed stressed plants of K-7 (**Figures 10** and **13**) can be attributed to the regulation of ROS levels by the action of melatonin thereby maintaining lipid peroxidation levels which is enough to provide the substrate for LOX activity. Additionally, our findings suggest that the expression of LOX2 is highest among all other isoforms, which also indicates its exigent role during osmotic stress as reported by Singh et al. (2022). The tolerant variety showcased higher LOX activity and content, which can be correlated to higher melatonin content and its stress tolerance nature. Surprisingly, lower LOX activity in primed stressed plants might be due to the lesser substrate availability. Therefore, endogenous melatonin content shares a direct relationship with LOX. Overall the study has shown the significant role of melatonin in modulating the expression of lipoxygenases under drought stress (**Figure 12B**).

Abiotic stress responses, including drought, are known to be mediated by jasmonic acid. The negative effects of drought-induced membrane damage in barley were found to be mitigated by JA pre-treatment (Bandurska et al., 2003). Wang et al. (2021) reported that the genes involved in JA biosynthesis and signaling were upregulated by drought priming during drought tolerance in wheat. In consistent with these reports, our studies also indicated that melatonin increased the jasmonic acid content (**Figure 11A**) and expression of genes (*LOX*, *AOS*, *AOC* and *OPDAR3*) involved in the jasmonic acid biosynthesis pathway under stress condition in K-7 (**Figure 12**; **Supplementary Figures 7** and **8A–C**). The JA levels in K-9 also indicate the direct relationship between LOX and JA under stress condition, which could be due to higher endogenous melatonin content.

ABA and JA are the key molecules required for stress tolerance response. Higher levels of JA were found to be essential in ABA accumulation during drought stress in *Arabidopsis* (de Ollas et al., 2015) and rice (Kim et al., 2009). In accordance with these studies, it was observed that melatonin enhanced JA biosynthesis *via* stimulating LOX which further promoted ABA production in K-7 under stress. However, in K-9, enhanced levels of ABA in unprimed stressed plants and a significant decrease in primed plants under stress correlated to JA content suggesting the involvement of JA on ABA accumulation under drought condition (**Figure 11B**). A correlative expression of Snf-related kinase protein (*SnRK2*) was observed in our study (**Supplementary Figure 9C**) which is responsible for ABA-mediated dehydration signaling response (Yoshida et al., 2002). These findings indicate that JA might function as an upstream modulator in ABA-mediated signaling, as suggested by Wang et al. (2021) and regulates the tolerance response under drought stress. Moreover, upregulated expression of the *MYC2* transcription factor has been reported to positively regulate the genes involved in the antioxidant defense system (Wang et al., 2021). In consistent with these reports, melatonin was found to enhance the transcript levels of MYC2 in K-7 under stress, thereby enhancing its drought stress tolerance (**Supplementary Figure 8D**).

**Figure 14:**
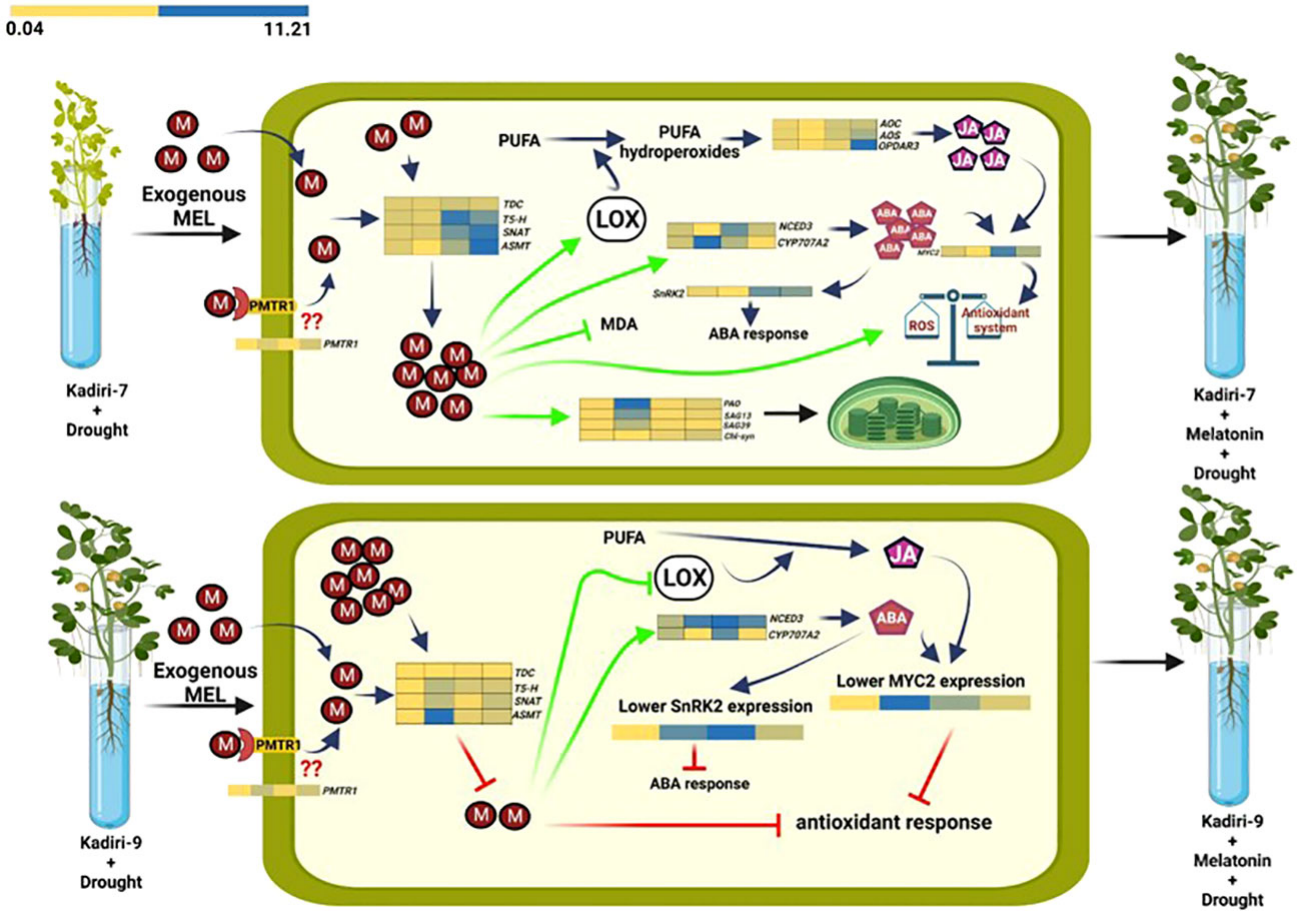
Differential effects of melatonin priming on drought-sensitive (Kadiri-7, upper) and drought-tolerant (Kadiri-9, lower) varieties of groundnut under drought stress to alleviate the impact of stress. Melatonin confers the tolerance in Kadiri-7 by improving endogenous melatonin content, antioxidant system and photosynthetic efficiency. Additionally, melatonin-mediated amelioration of LOX expression and activity in turn increases the endogenous JA and ABA contents, which also provides stress tolerance. Furthermore, unprimed Kadiri-9 with higher endogenous melatonin correlates with its inherent tolerance nature. Contrarily, exogenous melatonin priming was found to decrease its endogenous level, possibly as a consequence of feedback inhibition to fine-tune melatonin biosynthesis and maintain redox homeostasis towards better plant metabolism under drought stress condition.

The model showing the regulatory differences of melatonin action between drought-sensitive (K-7) and drought-tolerant (K-9) varieties under drought stress is depicted in **Figure 14** (this image is made using BioRender).

## 5 Conclusion

Melatonin is well known for its potential to impart drought stress tolerance in different plant species due to its capability to scavenge ROS, enhance photosynthetic efficiency and modulate the expression of stress-responsive genes. The virtue of exogenous melatonin on drought tolerance might be different in sensitive and tolerant varieties as their redox state differs under drought stress conditions. The present study for the first time reports the differential effects exhibited by exogenous melatonin on PEG-induced water stress attributes in the tolerant and sensitive varieties of groundnut. Melatonin priming improved drought tolerance by regulating redox homeostasis, promoting photosynthetic efficiency and increasing the chlorophyll content in sensitive variety. Additionally, melatonin-primed stressed plants of sensitive variety showed higher endogenous melatonin content with increased LOX expression, accompanied by elevated JA and ABA levels that could have imparted drought stress tolerance. However, melatonin priming led to a decrease in endogenous melatonin content in the tolerant variety under stress conditions possibly due to feedback inhibition to fine-tune melatonin biosynthesis required for maintaining ROS homeostasis towards better plant metabolism. The higher endogenous melatonin content in unprimed stressed plants of tolerant variety was associated with the enhanced antioxidant system, photosynthetic efficiency and LOX expression along with higher JA and ABA, which further substantiates the role of melatonin in drought stress tolerance. Overall, it can be concluded that exogenous melatonin evokes drought stress responses differently in sensitive and tolerant varieties as reflected by its ability to improve the tolerance of sensitive variety where stress effects are more prevalent in comparison to the tolerant variety that is inherently capable to tolerate the stress treatment given in the study. Thus, the study has advanced our scientific knowledge on the effects of exogenous melatonin with respect to modulation of LOX expression, JA and ABA which provides scope for improving the drought stress tolerance in groundnut.

## Data availability statement

The original contributions presented in the study are included in the article/**Supplementary Material**. Further inquiries can be directed to the corresponding author.

## Author contributions

GP, SS and LS: conceived and designed the study. SS and LS: conducted the experiments, collected the data, prepared the graphs, analyzed and interpreted the results and written the manuscript. GP: supervised, analyzed and interpreted the results and corrected the manuscript. All authors contributed to the article and approved the submitted version.

## Funding

GP gratefully acknowledges the research grant of Institute of Eminence (IoE) of UGC (UoH-IoE-RC3-21-041) sanctioned by University of Hyderabad which was used partly to carry out the research work.

## Acknowledgments

The infrastructural facilities established in the Department/School with the support of UGC-SAP-DRS-1 (Level-1, Phase-1), DST-FIST-Level-II (Phase-2), DST-PURSE and DBT-BUILDER programmes have been utilized for the research work which is gratefully acknowledged. We are highly grateful to Dr. K. S. S. Naik, Principal Scientist (Groundnut) & Head, Agricultural Research Station, Acharya N. G. Ranga Agricultural University (ANGRAU), Kadiri, Anantapur, Andhra Pradesh for providing seeds of Kadiri-7 and Kadiri-9 varieties, used in this research work. We express our grateful thanks to Prof. S. Rajagopal, Department of Plant Sciences, School of Life Sciences, University of Hyderabad for his help in carrying the experiments of photosynthesis. Our grateful thanks to Dr. M. Muthamilarasan, Department of Plant Sciences, School of Life Sciences, University of Hyderabad for his helpful suggestions during the study. Thanks to Mr. Arpan Chatterjee, Department of Biochemistry, University of Hyderabad for helping out in immunoblot analysis. Mr. Ayon Chatterjee, Department of Biochemistry, University of Hyderabad has helped in HPLC analysis, which is gratefully acknowledged.

## Conflict of interest

The authors declare that the research was conducted in the absence of any commercial or financial relationships that could be construed as a potential conflict of interest.

## Publisher’s note

All claims expressed in this article are solely those of the authors and do not necessarily represent those of their affiliated organizations, or those of the publisher, the editors and the reviewers. Any product that may be evaluated in this article, or claim that may be made by its manufacturer, is not guaranteed or endorsed by the publisher.
