# Supplementary material for: Melatonin induces drought tolerance by modulating lipoxygenase expression, redox homeostasis and photosynthetic efficiency in Arachis hypogaea L
Source: Front Plant Sci. 2022 Dec 5;13:1069143. doi: 10.3389/fpls.2022.1069143 (PMC9760964; doi:10.3389/fpls.2022.1069143)
Supplement: Supplementary file 1 [file Presentation_1.pptx]

## Slide 1
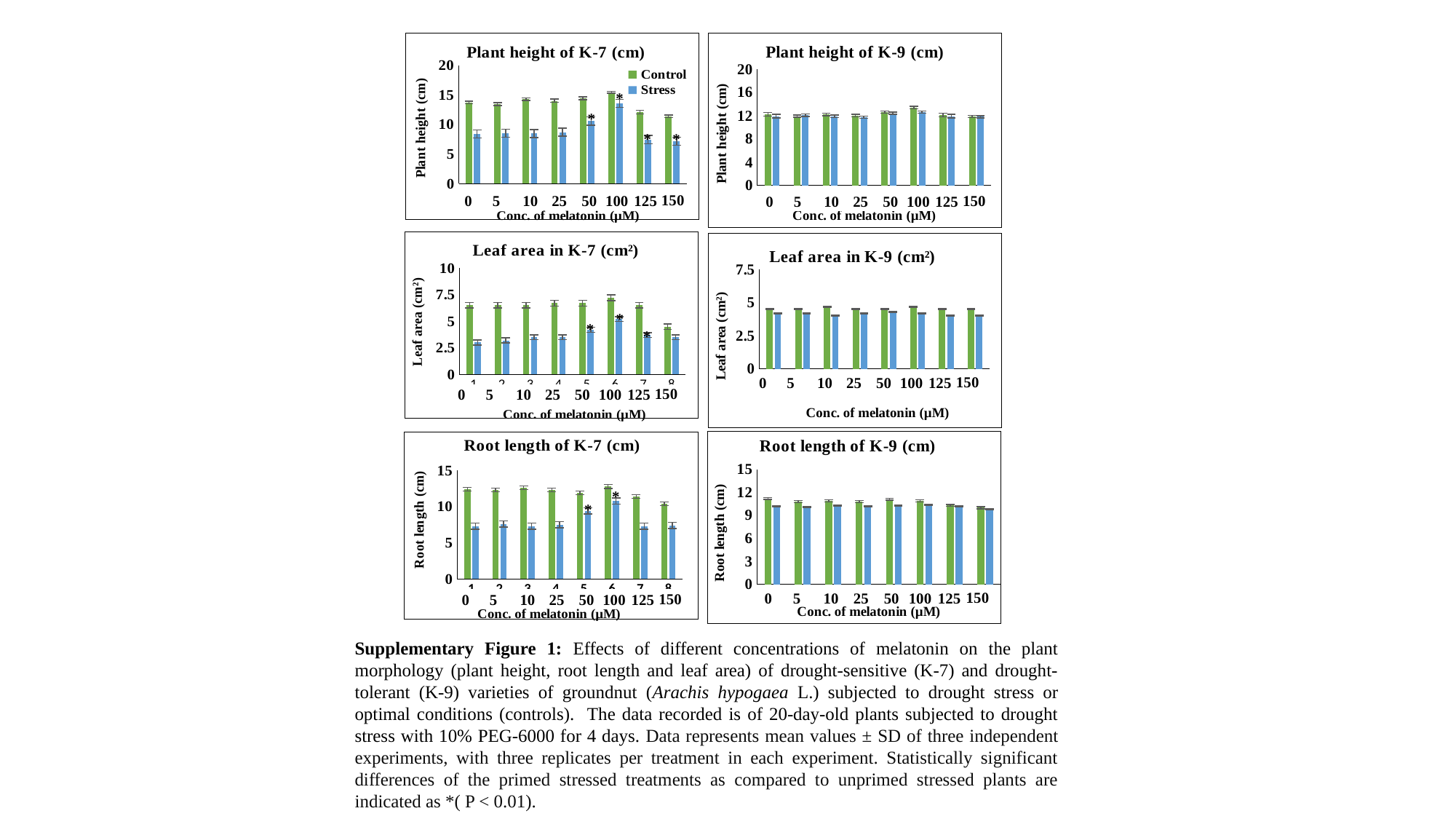

### Chart: Plant height of K-7 (cm)
| Category | Control | Stress |
|---|---|---|150
100
125
25
10
0
5
50
*
*
*
*
### Chart: Plant height of K-9 (cm)
| Category | Control | Str |
|---|---|---|150
100
125
25
10
0
5
50
### Chart: Leaf area in K-7 (cm²)
| Category | Control | Stress |
|---|---|---|150
100
125
25
10
0
5
50
*
*
*
### Chart: Leaf area in K-9 (cm²)
| Category | Control | Stress |
|---|---|---|150
100
125
25
10
0
5
50
### Chart: Root length of K-9 (cm)
| Category | Control | Stress |
|---|---|---|150
100
125
25
10
0
5
50
### Chart: Root length of K-7 (cm)
| Category | Control | Stress |
|---|---|---|150
100
125
25
10
0
5
50
*
*
Supplementary Figure 1: Effects of different concentrations of melatonin on the plant morphology (plant height, root length and leaf area) of drought-sensitive (K-7) and drought-tolerant (K-9) varieties of groundnut (Arachis hypogaea L.) subjected to drought stress or optimal conditions (controls). The data recorded is of 20-day-old plants subjected to drought stress with 10% PEG-6000 for 4 days. Data represents mean values ± SD of three independent experiments, with three replicates per treatment in each experiment. Statistically significant differences of the primed stressed treatments as compared to unprimed stressed plants are indicated as *( P < 0.01).

## Slide 2
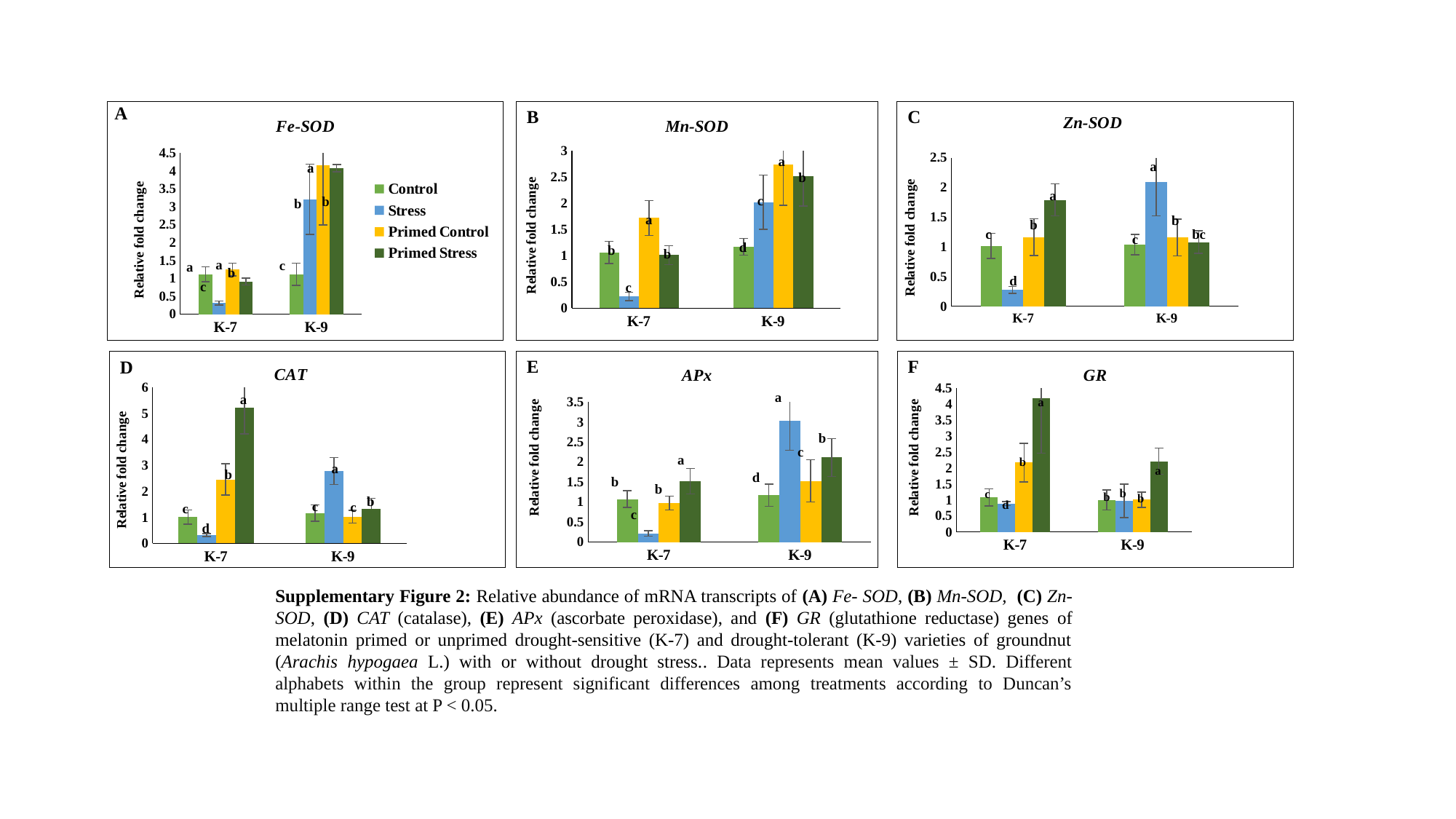

A
### Chart: Fe-SOD
| Category | Control | Stress | Primed Control | Primed Stress |
|---|---|---|---|---|
| K-7 | 1.117 | 0.314 | 1.246 | 0.912 |
| K-9 | 1.114 | 3.21 | 4.16 | 4.08 |a
b
b
a
c
a
b
c
### Chart: Mn-SOD
| Category | Control | Stress | Primed Control | Primed Stress |
|---|---|---|---|---|
| K-7 | 1.062 | 0.221 | 1.719 | 1.012 |
| K-9 | 1.17 | 2.02 | 2.74 | 2.51 |a
b
c
a
d
b
b
c
### Chart: Zn-SOD
| Category | Control | Stress | Primed Control | Primed Stress |
|---|---|---|---|---|
| K-7 | 1.017 | 0.276 | 1.167 | 1.79 |
| K-9 | 1.04 | 2.09 | 1.16 | 1.08 |a
a
b
b
c
bc
c
d
### Chart: CAT
| Category | Control | Stress | Primed Control | Primed Stress |
|---|---|---|---|---|
| K-7 | 1.014 | 0.321 | 2.46 | 5.214 |
| K-9 | 1.17 | 2.781 | 1.029 | 1.32 |
### Chart: APx
| Category | Control | Stress | Primed Control | Primed Stress |
|---|---|---|---|---|
| K-7 | 1.07 | 0.214 | 0.974 | 1.52 |
| K-9 | 1.17 | 3.04 | 1.53 | 2.1147 |
### Chart: GR
| Category | Control | Stress | Primed Control | Primed Stress |
|---|---|---|---|---|
| K-7 | 1.08 | 0.89 | 2.17 | 4.17 |
| K-9 | 1.003 | 0.97 | 1.007 | 2.21 |a
a
a
b
c
a
b
a
a
b
d
b
b
b
c
b
b
b
d
c
c
c
c
d
Supplementary Figure 2: Relative abundance of mRNA transcripts of (A) Fe- SOD, (B) Mn-SOD, (C) Zn-SOD, (D) CAT (catalase), (E) APx (ascorbate peroxidase), and (F) GR (glutathione reductase) genes of melatonin primed or unprimed drought-sensitive (K-7) and drought-tolerant (K-9) varieties of groundnut (Arachis hypogaea L.) with or without drought stress.. Data represents mean values ± SD. Different alphabets within the group represent significant differences among treatments according to Duncan’s multiple range test at P ˂ 0.05.

## Slide 3
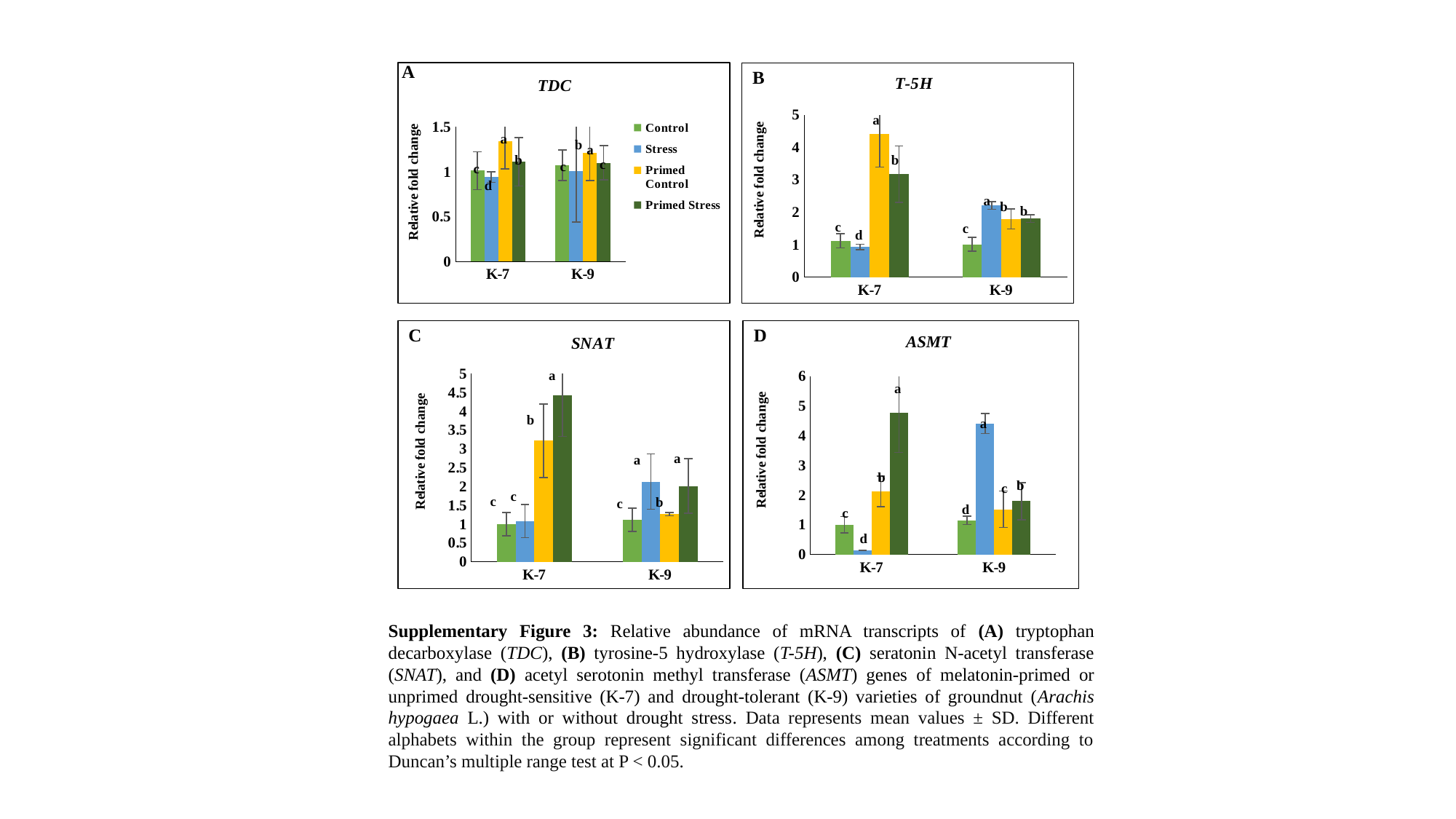

A
### Chart: TDC
| Category | Control | Stress | Primed Control | Primed Stress |
|---|---|---|---|---|
| K-7 | 1.013 | 0.941 | 1.341 | 1.112 |
| K-9 | 1.072 | 1.01 | 1.213 | 1.1 |a
b
a
b
c
c
c
d
### Chart: T-5H
| Category | Control | Stress | Primed Control | Primed Stress |
|---|---|---|---|---|
| K-7 | 1.12 | 0.929 | 4.417 | 3.17 |
| K-9 | 1.014 | 2.212 | 1.794 | 1.803 |a
b
a
b
b
c
c
d
### Chart: SNAT
| Category | Control | Stress | Primed Control | Primed Stress |
|---|---|---|---|---|
| K-7 | 1.0014 | 1.079 | 3.214 | 4.416 |
| K-9 | 1.12 | 2.13 | 1.267 | 2.012 |
### Chart: ASMT
| Category | Control | Stress | Primed Control | Primed Stress |
|---|---|---|---|---|
| K-7 | 1.0018 | 0.142 | 2.12 | 4.78 |
| K-9 | 1.146 | 4.41 | 1.52 | 1.792 |Supplementary Figure 3: Relative abundance of mRNA transcripts of (A) tryptophan decarboxylase (TDC), (B) tyrosine-5 hydroxylase (T-5H), (C) seratonin N-acetyl transferase (SNAT), and (D) acetyl serotonin methyl transferase (ASMT) genes of melatonin-primed or unprimed drought-sensitive (K-7) and drought-tolerant (K-9) varieties of groundnut (Arachis hypogaea L.) with or without drought stress. Data represents mean values ± SD. Different alphabets within the group represent significant differences among treatments according to Duncan’s multiple range test at P ˂ 0.05.

## Slide 4
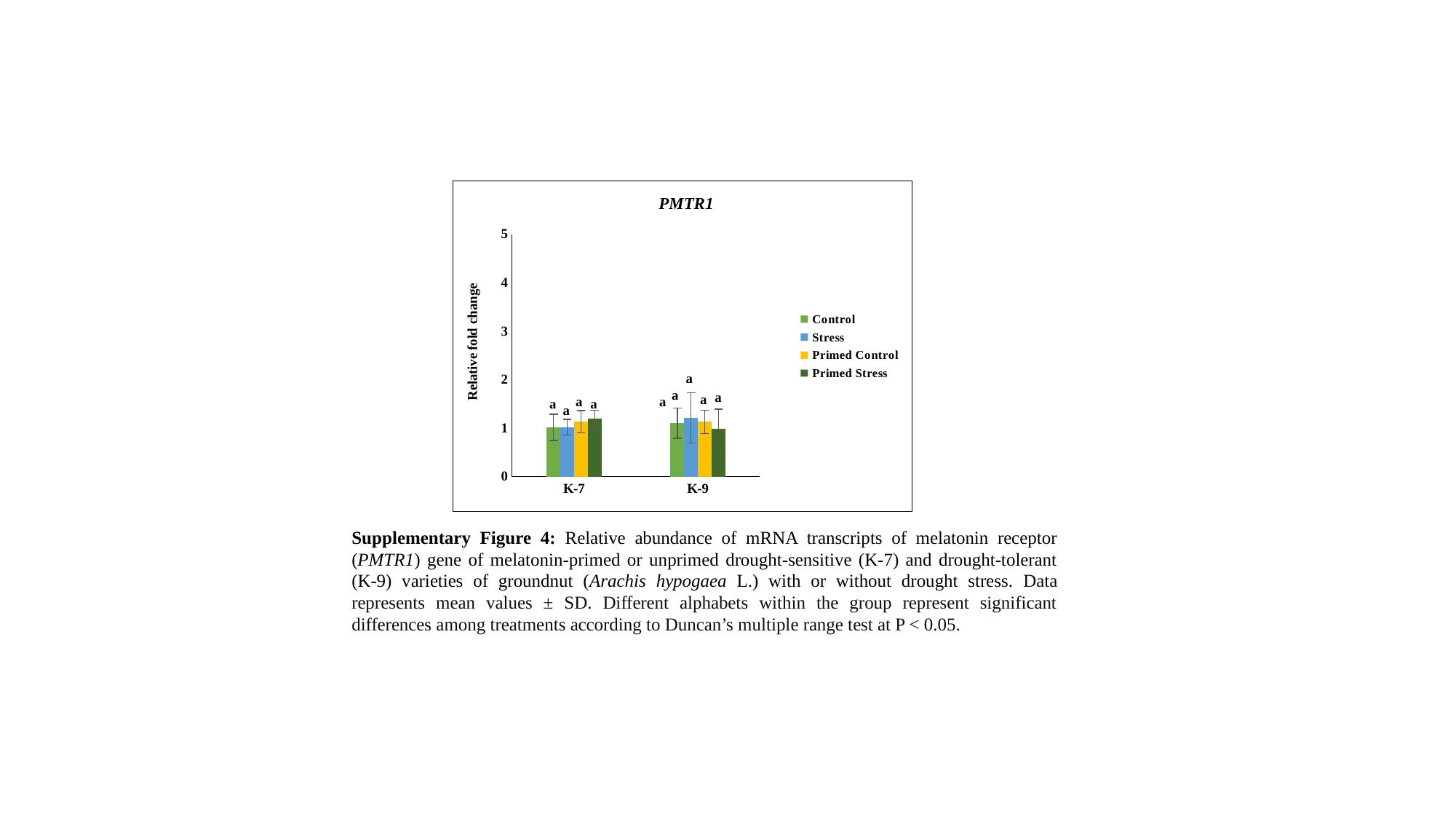

### Chart: PMTR1
| Category | Control | Stress | Primed Control | Primed Stress |
|---|---|---|---|---|
| K-7 | 1.012 | 1.02 | 1.13 | 1.2 |
| K-9 | 1.1 | 1.21 | 1.13 | 0.98 |Supplementary Figure 4: Relative abundance of mRNA transcripts of melatonin receptor (PMTR1) gene of melatonin-primed or unprimed drought-sensitive (K-7) and drought-tolerant (K-9) varieties of groundnut (Arachis hypogaea L.) with or without drought stress. Data represents mean values ± SD. Different alphabets within the group represent significant differences among treatments according to Duncan’s multiple range test at P ˂ 0.05.

## Slide 5
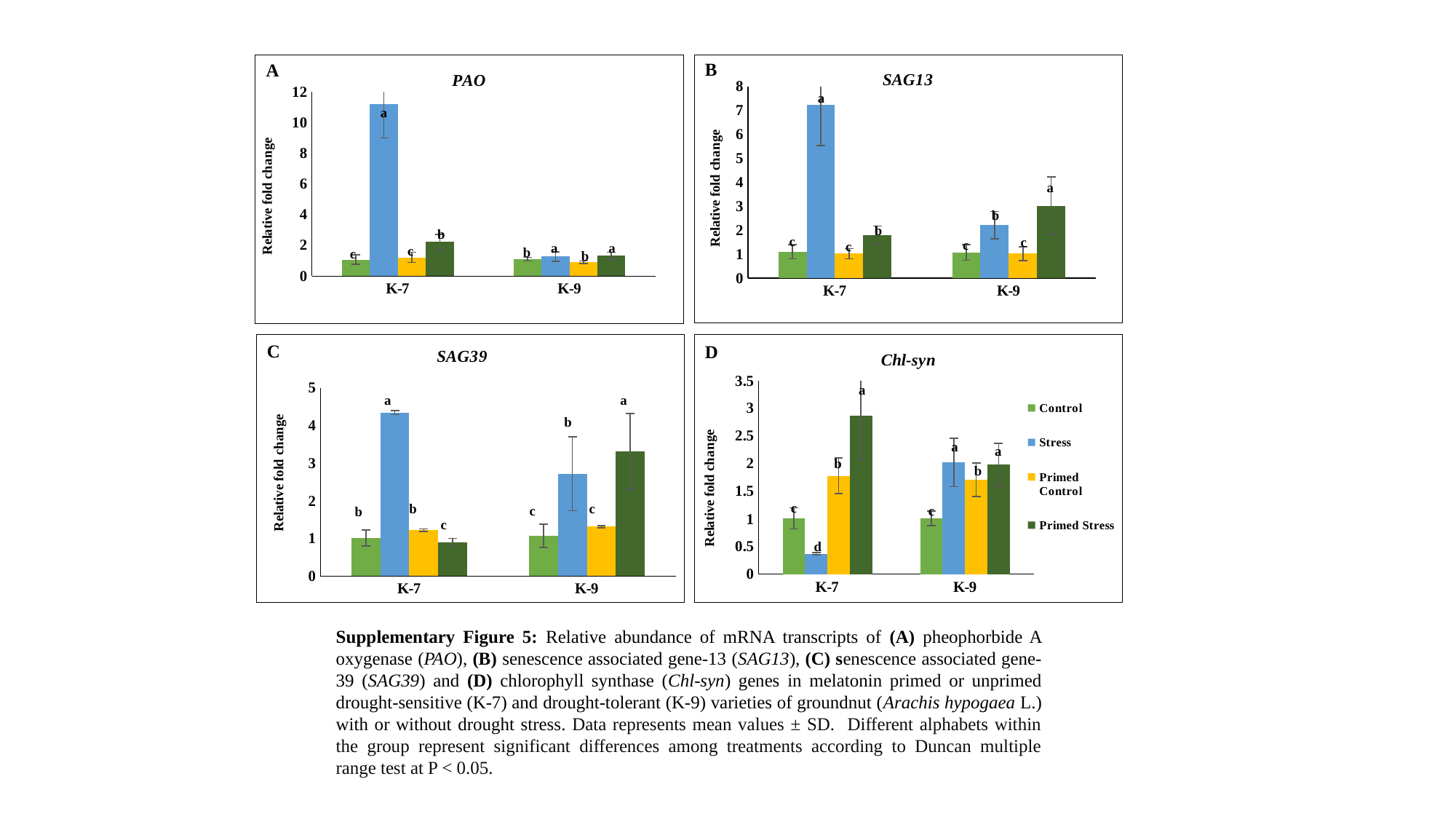

### Chart: SAG13
| Category | Control | Stress | Primed Control | Primed Stress |
|---|---|---|---|---|
| K-7 | 1.1 | 7.22 | 1.02 | 1.8 |
| K-9 | 1.07 | 2.21 | 1.02 | 3.02 |a
a
b
b
c
c
c
c
### Chart: PAO
| Category | Control | Stress | Primed Control | Primed Stress |
|---|---|---|---|---|
| K-7 | 1.07 | 11.21 | 1.21 | 2.21 |
| K-9 | 1.1 | 1.27 | 0.89 | 1.32 |a
b
a
a
c
b
c
b
### Chart: SAG39
| Category | Control | Stress | Primed Control | Primed Stress |
|---|---|---|---|---|
| K-7 | 1.01 | 4.34 | 1.22 | 0.901 |
| K-9 | 1.07 | 2.72 | 1.31 | 3.31 |a
a
b
c
b
c
b
c
### Chart: Chl-syn
| Category | Control | Stress | Primed Control | Primed Stress |
|---|---|---|---|---|
| K-7 | 1.012 | 0.371 | 1.78 | 2.86 |
| K-9 | 1.01 | 2.02 | 1.71 | 1.98 |D
a
a
a
b
b
c
c
d
Supplementary Figure 5: Relative abundance of mRNA transcripts of (A) pheophorbide A oxygenase (PAO), (B) senescence associated gene-13 (SAG13), (C) senescence associated gene-39 (SAG39) and (D) chlorophyll synthase (Chl-syn) genes in melatonin primed or unprimed drought-sensitive (K-7) and drought-tolerant (K-9) varieties of groundnut (Arachis hypogaea L.) with or without drought stress. Data represents mean values ± SD. Different alphabets within the group represent significant differences among treatments according to Duncan multiple range test at P ˂ 0.05.

## Slide 6
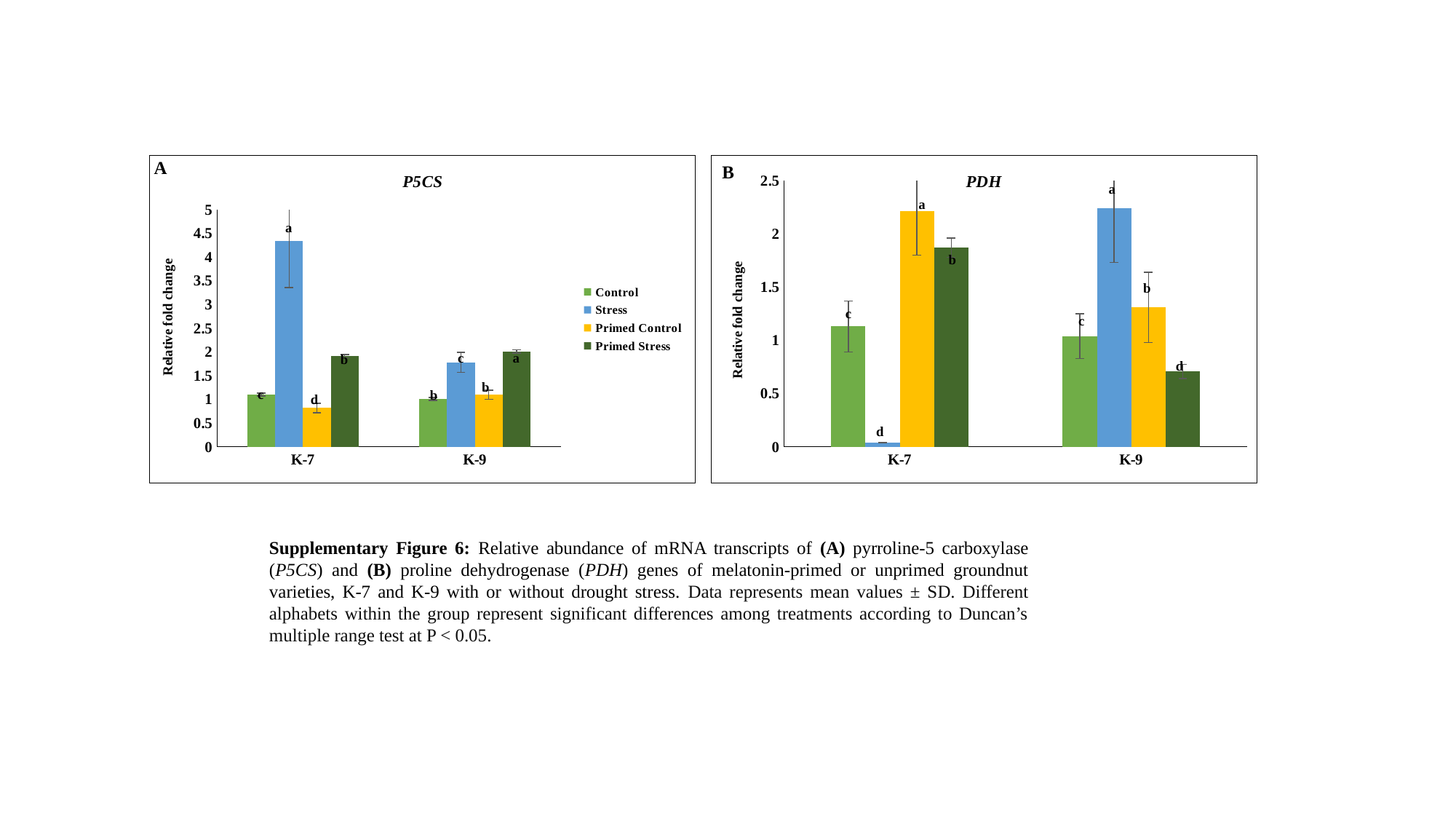

A
### Chart: P5CS
| Category | Control | Stress | Primed Control | Primed Stress |
|---|---|---|---|---|
| K-7 | 1.1 | 4.34 | 0.82 | 1.91 |
| K-9 | 1.01 | 1.78 | 1.1 | 2.01 |
### Chart: PDH
| Category | Control | Stress | Primed Control | Primed Stress |
|---|---|---|---|---|
| K-7 | 1.13 | 0.04 | 2.21 | 1.87 |
| K-9 | 1.04 | 2.24 | 1.31 | 0.708 |a
b
c
Supplementary Figure 6: Relative abundance of mRNA transcripts of (A) pyrroline-5 carboxylase (P5CS) and (B) proline dehydrogenase (PDH) genes of melatonin-primed or unprimed groundnut varieties, K-7 and K-9 with or without drought stress. Data represents mean values ± SD. Different alphabets within the group represent significant differences among treatments according to Duncan’s multiple range test at P ˂ 0.05.

## Slide 7
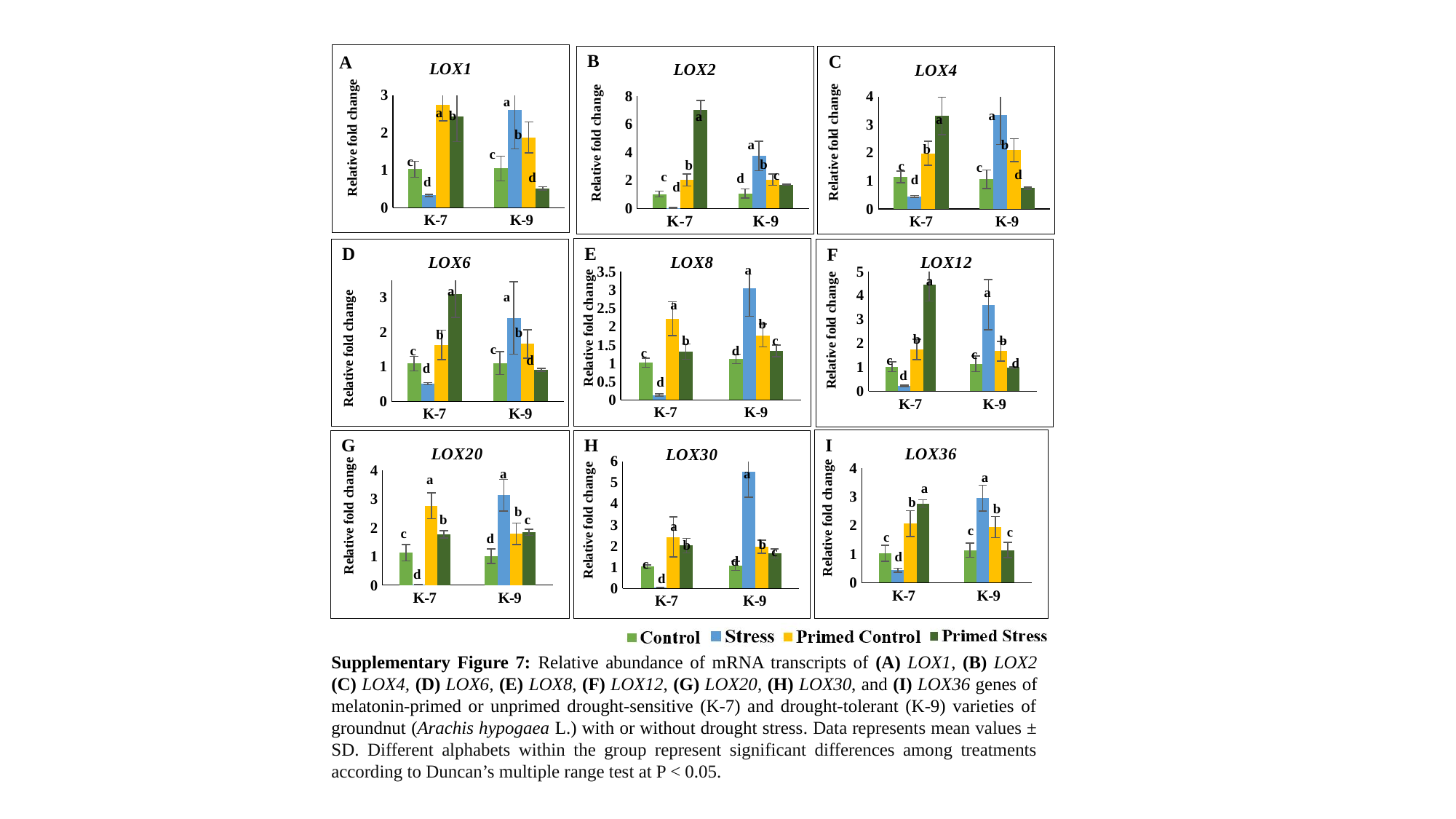

A
### Chart: LOX1
| Category | Control | Stress | Primed Control | Primed Stress |
|---|---|---|---|---|
| K-7 | 1.02 | 0.321 | 2.74 | 2.43 |
| K-9 | 1.04 | 2.61 | 1.87 | 0.51 |a
a
b
b
c
c
d
d
### Chart: LOX2
| Category | Control | Stress | Primed Control | Primed Stress |
|---|---|---|---|---|
| K-7 | 1.03 | 0.041 | 2.03 | 7.03 |
| K-9 | 1.07 | 3.741 | 2.06 | 1.69 |a
a
b
b
c
c
d
d
### Chart: LOX4
| Category | Control | Stress | Primed Control | Primed Stress |
|---|---|---|---|---|
| K-7 | 1.14 | 0.442 | 1.98 | 3.31 |
| K-9 | 1.05 | 3.34 | 2.09 | 0.74 |a
a
b
b
c
c
d
d
### Chart: LOX8
| Category | Control | Stress | Primed Control | Primed Stress |
|---|---|---|---|---|
| K-7 | 1.014 | 0.131 | 2.221 | 1.32 |
| K-9 | 1.12 | 3.04 | 1.76 | 1.34 |a
a
b
b
c
d
c
d
### Chart: LOX6
| Category | Control | Stress | Primed Control | Primed Stress |
|---|---|---|---|---|
| K-7 | 1.09 | 0.51 | 1.63 | 3.09 |
| K-9 | 1.1 | 2.41 | 1.66 | 0.91 |a
a
b
b
c
c
d
d
### Chart: LOX12
| Category | Control | Stress | Primed Control | Primed Stress |
|---|---|---|---|---|
| K-7 | 1.02 | 0.221 | 1.74 | 4.43 |
| K-9 | 1.14 | 3.61 | 1.67 | 0.98 |a
a
b
b
c
c
d
d
### Chart: LOX36
| Category | Control | Stress | Primed Control | Primed Stress |
|---|---|---|---|---|
| K-7 | 1.03 | 0.43 | 2.07 | 2.77 |
| K-9 | 1.14 | 2.96 | 1.94 | 1.14 |a
a
b
b
c
c
c
d
### Chart: LOX20
| Category | Control | Stress | Primed Control | Primed Stress |
|---|---|---|---|---|
| K-7 | 1.13 | 0.03 | 2.77 | 1.77 |
| K-9 | 1.01 | 3.13 | 1.79 | 1.84 |a
a
b
b
c
c
d
d
### Chart: LOX30
| Category | Control | Stress | Primed Control | Primed Stress |
|---|---|---|---|---|
| K-7 | 1.03 | 0.044 | 2.43 | 2.02 |
| K-9 | 1.06 | 5.52 | 1.97 | 1.65 |a
a
b
b
c
d
c
d
Supplementary Figure 7: Relative abundance of mRNA transcripts of (A) LOX1, (B) LOX2 (C) LOX4, (D) LOX6, (E) LOX8, (F) LOX12, (G) LOX20, (H) LOX30, and (I) LOX36 genes of melatonin-primed or unprimed drought-sensitive (K-7) and drought-tolerant (K-9) varieties of groundnut (Arachis hypogaea L.) with or without drought stress. Data represents mean values ± SD. Different alphabets within the group represent significant differences among treatments according to Duncan’s multiple range test at P ˂ 0.05.

## Slide 8
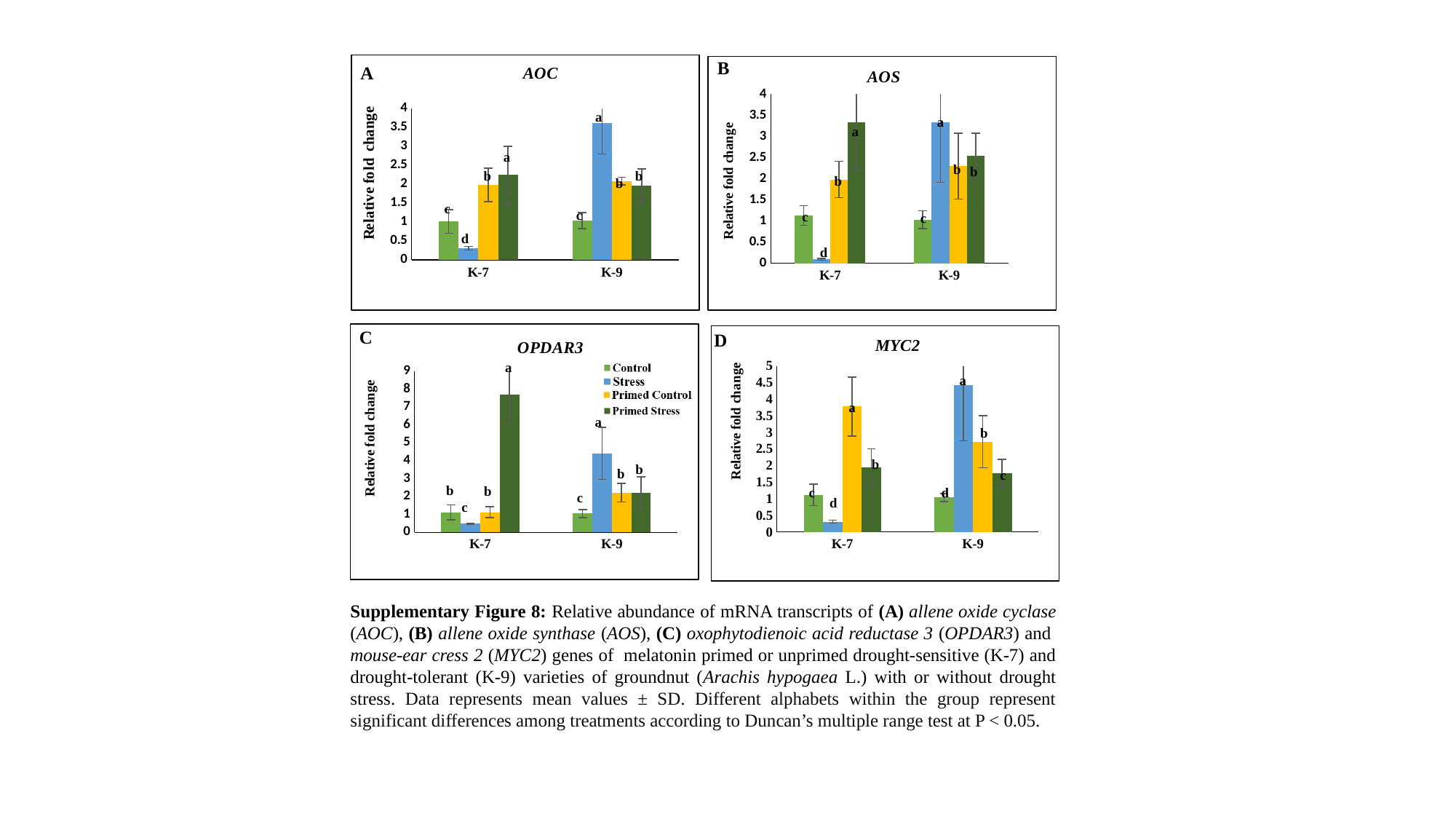

B
### Chart: AOC
| Category | Control | Stress | Primed Control | Primed Stress |
|---|---|---|---|---|
| K-7 | 1.012 | 0.31 | 1.98 | 2.24 |
| K-9 | 1.04 | 3.61 | 2.08 | 1.96 |a
a
b
b
b
c
c
d
A
### Chart: AOS
| Category | Control | Stress | Primed Control | Primed Stress |
|---|---|---|---|---|
| K-7 | 1.13 | 0.091 | 1.98 | 3.34 |
| K-9 | 1.03 | 3.34 | 2.3 | 2.54 |a
a
b
b
b
c
c
d
C
### Chart: OPDAR3
| Category | Control | Stress | Primed Control | Primed Stress |
|---|---|---|---|---|
| K-7 | 1.11 | 0.48 | 1.119 | 7.71 |
| K-9 | 1.04 | 4.41 | 2.21 | 2.21 |a
b
b
b
b
c
c
D
### Chart: MYC2
| Category | Control | Stress | Primed Control | Primed Stress |
|---|---|---|---|---|
| K-7 | 1.12 | 0.312 | 3.78 | 1.94 |
| K-9 | 1.04 | 4.42 | 2.72 | 1.76 |a
a
b
b
c
c
d
d
a
Supplementary Figure 8: Relative abundance of mRNA transcripts of (A) allene oxide cyclase (AOC), (B) allene oxide synthase (AOS), (C) oxophytodienoic acid reductase 3 (OPDAR3) and mouse-ear cress 2 (MYC2) genes of melatonin primed or unprimed drought-sensitive (K-7) and drought-tolerant (K-9) varieties of groundnut (Arachis hypogaea L.) with or without drought stress. Data represents mean values ± SD. Different alphabets within the group represent significant differences among treatments according to Duncan’s multiple range test at P ˂ 0.05.

## Slide 9
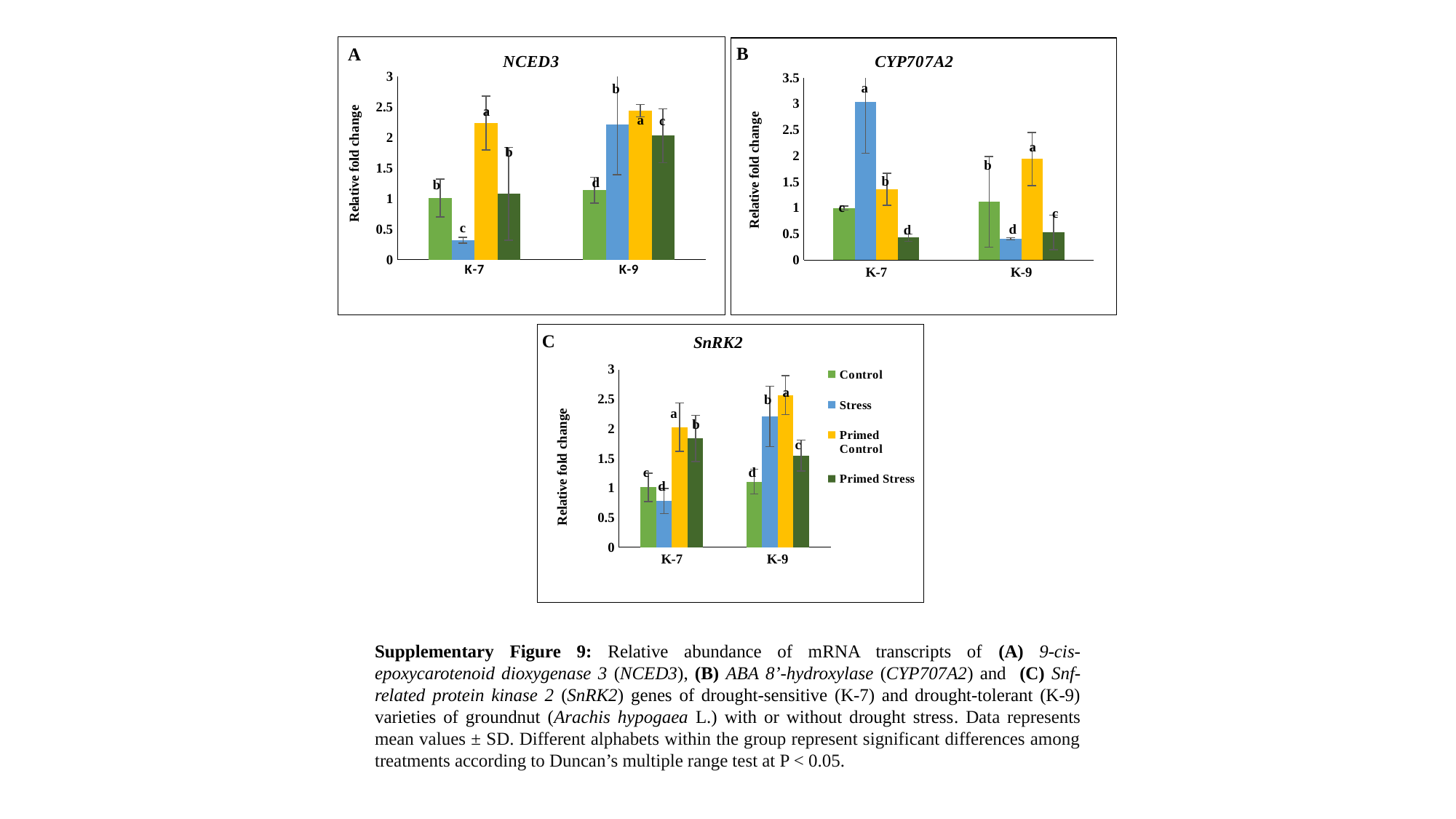

### Chart: NCED3
| Category | Control | Stress | Primed Control | Primed Stress |
|---|---|---|---|---|
| K-7 | 1.012 | 0.32 | 2.24 | 1.08 |
| K-9 | 1.14 | 2.21 | 2.44 | 2.03 |b
a
a
c
b
d
b
c
### Chart: CYP707A2
| Category | Control | Stress | Primed Control | Primed Stress |
|---|---|---|---|---|
| K-7 | 1.001 | 3.032 | 1.36 | 0.43 |
| K-9 | 1.12 | 0.41 | 1.94 | 0.53 |a
a
b
b
c
c
d
d
### Chart: SnRK2
| Category | Control | Stress | Primed Control | Primed Stress |
|---|---|---|---|---|
| K-7 | 1.014 | 0.782 | 2.03 | 1.84 |
| K-9 | 1.11 | 2.21 | 2.57 | 1.55 |a
b
a
b
c
c
d
d
B
A
C
Supplementary Figure 9: Relative abundance of mRNA transcripts of (A) 9-cis-epoxycarotenoid dioxygenase 3 (NCED3), (B) ABA 8’-hydroxylase (CYP707A2) and (C) Snf-related protein kinase 2 (SnRK2) genes of drought-sensitive (K-7) and drought-tolerant (K-9) varieties of groundnut (Arachis hypogaea L.) with or without drought stress. Data represents mean values ± SD. Different alphabets within the group represent significant differences among treatments according to Duncan’s multiple range test at P ˂ 0.05.
