# Supplementary material for: Melatonin induces drought tolerance by modulating lipoxygenase expression, redox homeostasis and photosynthetic efficiency in Arachis hypogaea L
Source: Front Plant Sci. 2022 Dec 5;13:1069143. doi: 10.3389/fpls.2022.1069143 (PMC9760964; doi:10.3389/fpls.2022.1069143)
Supplement: Supplementary file 2 [file Table_1.docx]

| **Name of the genes** | **Forward primer (5’ to 3’)** | **Reverse primer (5’ to 3’)** |
| --- | --- | --- |
| *Actin* | TGATCGGGATGGAATCTC | CCACCACTCAAGACAATG |
| *Fe-SOD* | TCAGCCCTCTGCATACTA | GGGTCCTCAAGCCATAAA |
| *Mn-SOD* | GCTGCCGGATCTGAATTA | TGATGGTGCTTCTTGTGG |
| *Cu/Zn-SOD* | CGGCCCTACTACTGTTAATG | ACAGCCGTTTGTGGTATC |
| *CAT* | AAGTCCAACGTCCAAGAG | ATGCCCACGTCATCATAG |
| *APx* | CGGCTTCCTGATGCTAAA | CTCCAGACAGTGCAACAA |
| *GR* | CAGCATTCCACCACTTTC | CGCCCTGAGATGGTATTT |
| *TDC* | ATGGTGATGCGTCTCTAC | TGAACCTTGGATCCTGAC |
| *T5-H* | CTCTTGCAATTCGGGCAC | TGGACTGTTGTCTTGGGA |
| *SNAT* | TCTGTGACAAGGTAGGGT | GACCCTGGAGACTTTCTTAC |
| *ASMT* | CCTTGGTGGGAGTTTCTT | CCCTCAAAGGCCATGTTA |
| *PMTR1* | CGCTCTCACATCATCATC | AGGAGAACACTCCCATAC |
| *PAO* | TCGTCTCGCTCCTTTATC | GCTTGAGGGATCCTAACA |
| *SAG13* | CACCAAGTCTCTCATCCA | AGAGTCCATCAGCTTCTC |
| *SAG39* | GATGCTAGTGGCTCTGAT | CTGACTCCATAACCCACA |
| *Chl-syn* | CACTCACACCCGACATTA | AAGAGACTGAAGCCCAAG |
| *P5CS* | GGGACAATGACAGTTTGG | GATGGAGGGCCACTATAA |
| *PDH* | GAATGAGGCGGGTTTAAG | GTGAAGGAAGCCGTTAAG |
| *LOX1* | CTCCAGCTCATCAGTTCT | GCTGAATGCCAGATTCTC |
| *LOX2* | GCGTCAGGAACATGAAAG | GGGTAAGGGAAAGTGGTA |
| *LOX4* | CGCAAAGAATGGGATAGG | TGCGAGGATAAGGATAGG |
| *LOX6* | CTTGCCAAGGCTTATGTC | GGATGGAGCACACTAAGA |
| *LOX8* | TAGCCAGGATGTGATTCC | CCGCCTTCATACAGATCA |
| *LOX12* | GCAACCGCCTGTTTATTC | GCGTTATCGCTCAGAAAC |
| *LOX20* | GGTGTATCCGAGCGATAA | CAGGCATTCCAGTTCTTC |
| *LOX30* | GCTGCTATACCCATCAGA | GTGCGGCGTTTCATAATC |
| *LOX36* | CGAGCGATACCGATATGT | CACGCTAAAGGTGTTCTG |
| *AOC* | AGCGAGTGGGAATAACAG | GGCCATAGTCTCCAAAGT |
| *AOS* | GAACCAAAGCACGAGAAG | CTCTGTCCAGGGAATCAA |
| *OPDAR3* | TTACGCTCAGAGATCCAC | GTTTCTCCATGCCTCAAC |
| *MYC2* | CTTCGGTGGAGGATGATT | GTGGCAGTGTTTGAGAAG |
| *NCED3* | GGAATGGTGAACCGAAAC | CTTAGCGAAACCCGAAAC |
| *CYP707A2* | GGAGGGTTATTGGGAGAA | GCCAAGTAAGAGCAGTTG |
| *SnRK2* | GGACCTGAAGTTGGAGAA | GAATGAAGCACCGAAGAC |

**Supplementary Table 1:** List of primer sequences of the genes used for analysing their transcript levels using real-time PCR
